# Use of procalcitonin point-of-care testing to guide de-escalation of antibiotic therapy in adults with suspected bacterial sepsis in a tertiary hospital in Bangladesh: a randomised controlled open-label trial

**DOI:** 10.1016/j.eclinm.2026.104171

**Published:** 2026-09-02

**Authors:** Forhad Uddin Hasan Chowdhury, Rabiul Alam Md Erfan Uddin, Mohammad Ali, Pranay Kumar Dutta, Durjoy Das, Abu Huraira Hanif, Stefano Di Gregorio, Moumita Kar, Faisal Bin Jahangir, Shafia Islam Khan, Karabi Chowdhury, Md Mohasin, Abdullah Abu Sayeed, Md Harunur Or Roshid, Naim Uddin Chy, Md Akram Hossain, Md Abdus Sattar, Marja Schilstra, Evelien de Jong, Constance Schultsz, Maarten Nijsten, Md Jashim Uddin, Aniruddha Ghose, Katherine Plewes, Luigi Pisani, Philippe Guerin, Mavuto Mukaka, Arjen M. Dondorp

**Affiliations:** a Centre for Tropical Medicine and Global Health, Nuffield Department of Medicine, University of Oxford, Oxford OX3 7FZ, United Kingdom; b Chattogram Medical College, K.B. Fazlul Kader Road, Chattogram 4203, Bangladesh; c University of Dhaka, Nilkhet Road, Dhaka 1000, Bangladesh; d University of Chittagong, Hathazari, Chattogram 4331, Bangladesh; e Apollo Imperial Hospitals, Zakir Hossain Road, Chattogram 4202, Bangladesh; f OLVG Hospital, 1091 AC Amsterdam, the Netherlands; g Amsterdam University Medical Centre, Meibergdreef 9, 1105 AZ Amsterdam, the Netherlands; h Amsterdam Institute of Global Health and Development, Paashevelweg 25, 1105 BP Amsterdam, the Netherlands; i University Medical Center Groningen, Hanzeplein 1, 9713 GZ Groningen, the Netherlands; j Mahidol Oxford Tropical Medicine Research Unit, Faculty of Tropical Medicine, Mahidol University, 420/6 Ratchawithi Road, Bangkok 10400, Thailand; k Division of Infectious Diseases, University of British Columbia, Vancouver, BC V6T 1Z4, Canada; l Infectious Diseases Data Observatory, University of Oxford, Oxford OX3 7FZ, United Kingdom; m Peter Medawar Building for Pathogen Research, University of Oxford, South Parks Road, Oxford, United Kingdom; n Department of Biochemistry and Molecular Biology, Bangladesh Medical University, Dhaka, Bangladesh; o University of Bari Aldo Moro, Bari, Italy

**Keywords:** Procalcitonin, Antibiotic de-escalation, Sepsis, Antimicrobial stewardship, Point-of-care testing, Bangladesh

## Abstract

**Background:**

Important drivers of antibiotic resistance in low- and middle-income countries (LMICs) include antibiotic overuse and scarcity of microbiological diagnostics. Procalcitonin point-of-care testing is a potential tool to guide antibiotic de-escalation in patients admitted with suspected bacterial sepsis.

**Methods:**

In an open-label randomised controlled clinical trial in Chittagong Medical College Hospital in Bangladesh, adult patients with suspected bacterial sepsis were randomised to procalcitonin (PCT)-guided de-escalation of antibiotic therapy or standard-of-care. In the intervention group, serum procalcitonin was assessed daily and clinicians received a non-binding advice to stop antibiotics after 72 h if procalcitonin dropped below pre-defined threshold concentrations (0.5 ng/mL or 80% from the peak value if baseline value > 2 ng/mL). The primary endpoint was length of antibiotic therapy analysed by intention-to-treat. The study is registered with ClinicalTrials.gov (NCT05955612) and was completed in July 2024.

**Findings:**

Between 26 July 2023 and 20 June 2024, 532 of the 1002 patients screened for eligibility were randomly assigned to procalcitonin-guided antibiotic therapy (n = 266) or standard-of-care (n = 266). Follow-up was completed on 30 July 2024. Median (interquartile range [IQR]) length of antibiotic therapy was 4.7 (2.8–8.0) days with procalcitonin-guided therapy compared to 9.0 (5.8–12.0) days in the control arm (p < 0.0001). Mortality was similar between the two groups. In the procalcitonin-guided arm, 23 of 265 participants died (8.7%; 95% confidence interval [CI], 5.6%–12.7%), compared with 21 of 265 participants (7.9%; 95% CI, 5.0%–11.9%) in the control arm. The absolute risk difference was 0.8 percentage points (95% CI, −3.9 to 5.5 percentage points) (p = 0.875). Following review, the Data Safety Monitoring Board did not identify any deaths as attributable to antibiotic discontinuation. The incidence of recurrent infections was 8/265 (3.0%) in procalcitonin-guided antibiotic therapy and 10/265 (3.8%) in the control arm (p = 0.811).

**Interpretation:**

In this single-centre trial in Bangladesh, procalcitonin-guided de-escalation reduced the duration of antibiotic therapy in adults with suspected bacterial sepsis. Mortality and recurrent infection were similar between the groups, although the trial was not powered to assess these outcomes formally. This promising approach of point-of-care procalcitonin-guided antibiotic discontinuation should be evaluated further in comparable resource-limited settings.

**Funding:**

Wellcome Trust of Great Britain.

**Research in context**

**Evidence before this study**

Several clinical trials from high-income countries (HICs) have evaluated dynamics of blood procalcitonin (PCT) concentrations as a tool to guide antibiotic therapy in patients with bacterial sepsis. The PRORATA trial from France demonstrated that PCT-guided de-escalation of antibiotic therapy in sepsis patients reduced antibiotic usage without increasing case fatality, while the SAPS trial in the Netherlands confirmed that this approach safely shortens antibiotic treatment duration in critically ill patients. More recently, the ADAPT-Sepsis trial in the UK showed that daily PCT monitoring safely reduced antibiotic usage, whereas guidance based on C-reactive protein (CRP) provided no clear advantage. Almost all clinical trials on PCT to guide antibiotic treatment are from HICs, which, compared with low- and middle-income countries (LMICs), have strong microbiological laboratory support and comparatively lower antimicrobial resistance (AMR) prevalence. A recent systematic review stated that evidence on the use of PCT from LMICs is limited and inconsistent. The Surviving Sepsis Campaign 2021 guidelines suggest using procalcitonin together with clinical evaluation for the timing of antimicrobial discontinuation in patients with sepsis or septic shock, but this recommendation is rated as weak and based on low-certainty evidence derived mainly from high-income intensive care unit (ICU) studies.

**Added value of this study**

To our knowledge, the PROCALBAN trial is among the first randomised controlled trials to evaluate procalcitonin-guided antibiotic de-escalation in adults with a clinical diagnosis of bacterial sepsis in a low-resource setting with a high burden of antimicrobial resistance, using an affordable point-of-care procalcitonin test. The study found that daily point-of-care assessment of serum procalcitonin concentrations to guide antibiotic de-escalation reduced the duration of antibiotic therapy compared with standard care. The study was conducted in both general medical wards and the intensive care unit, reflecting sepsis management at the study hospital. These findings add evidence from a low-resource setting, where data on procalcitonin-guided antibiotic de-escalation remain limited.

**Implications of all the available evidence**

Evidence from high-income settings and this trial suggests that procalcitonin-guided de-escalation can safely reduce antibiotic duration in adults with suspected bacterial sepsis. Additional studies from other LMICs are needed before a more generalised recommendation covering all settings can be provided.

## Introduction

Overuse of antibiotics to treat febrile illnesses is a global problem at all levels of the health system, but antibiotic overuse is more prominent in low- and middle-income countries (LMICs).^1^ Antibiotic overuse is an important problem in hospitalised patients with critical illness, including sepsis.^2^ The contribution of sepsis to the overall disease burden is disproportionately high in countries with a low-to-middle socio-demographic index, accounting for roughly 85% of the global number of sepsis cases and deaths.^3^

Early initiation of antibiotic therapy in sepsis patients saves lives, but excessive use of antibiotics increases drug pressure driving antimicrobial resistance (AMR).^4^ According to global estimates, antibiotic resistant bacterial infections were responsible for approximately 4.95 million deaths in 2019.^5^

Timely discontinuation of antibiotics is a major challenge in the management of sepsis, particularly in low-resourced settings, where access to quality microbiology laboratories to guide antibiotic treatment is limited.^6^^,^^7^ White blood cell (WBC) counts and blood C-reactive protein (CRP) concentrations have been used to guide antibiotic de-escalation, including in Bangladesh, but these lack predictive accuracy to define microbiological cure.^8^ The dynamics of blood procalcitonin (PCT) concentrations after starting antibiotic treatment is a more promising tool to guide de-escalation of antibiotic therapy in sepsis.^9^ In bacterial sepsis, PCT is released mainly by mononuclear cells in response to bacterial endotoxins and pro-inflammatory cytokines.^10^ Randomised controlled trials from high-income countries (HICs) show that antibiotics can be discontinued safely when blood PCT concentrations drop below a predefined threshold.^11^ Most of these studies have been conducted in intensive care units (ICU),^11^^,^^12^ whereas in LMICs, where ICU capacity is limited, most sepsis patients are managed in general wards. High costs of PCT testing have also been a barrier for its deployment in LMICs, but recent introduction of affordable rapid point-of-care PCT assays has made PCT testing more feasible in LMICs.^13^

The objective of this trial was to evaluate the effectiveness and safety of procalcitonin-guided de-escalation of antibiotics in a randomised controlled trial in adult patients with sepsis admitted to Chittagong Medical College Hospital (CMCH), a tertiary hospital in Chattogram, Bangladesh.

## Methods

### Study design

This PROCALBAN study was a prospective, single-centre, randomised, parallel-group, open-label superiority trial conducted in patients admitted to Chittagong Medical College Hospital (CMCH), a tertiary care referral hospital in Chattogram, Bangladesh. The trial was designed to assess whether procalcitonin-guided antibiotic de-escalation reduced the length of antibiotic therapy compared with standard of care. The trial protocol and Statistical Analysis Plan are available through the ClinicalTrials.gov trial registration record, NCT05955612. This trial is reported in accordance with the CONSORT 2025 statement.^14^

### Participants

Eligible participants were aged 16–65 years, recruited within 24 h of admission to the general medicine wards or ICU with confirmed or suspected bacterial sepsis, an intention to receive parenteral antibiotic therapy and a National Early Warning Score (NEWS) of ≥5 or a single NEWS criterion ≥3.^15^ SOFA score was not used as an eligibility criterion. Modified SOFA was calculated retrospectively to describe baseline organ dysfunction; full conventional SOFA could not be calculated because arterial blood gas measurements were not routinely available, and the respiratory component was therefore estimated using the SpO_2_/FiO_2_ ratio. Exclusion criteria included: suspected or confirmed tuberculosis, documented parasitic or viral infection, pregnancy, anticipated short ICU or ward stays (e.g., post-operative cases), infections requiring pre-defined prolonged antibiotic therapy (e.g., osteomyelitis or infective endocarditis), documented immunocompromised status, prior parenteral antibiotic use exceeding 48 h at the moment of screening, infection requiring surgical source control, moribund patients or patients receiving end-of-life care, and previously enrolled participants in the study. Race and ethnicity data were not collected because these variables were not prespecified in the trial dataset for this single-centre study in Bangladesh.

### Ethics

The study protocol was approved by the Oxford Tropical Research Ethics Committee, University of Oxford (OxTREC; 12–23), the Bangladesh Medical Research Council (BMRC/NREC/2022-2025/151; 532 04 04 2023), and the Ethics Review Committee of Chattogram Medical College (ERC; 59.27.0000.013.19.PG.2023.009/280). Written informed consent was obtained from all participants or, where applicable, their legal representatives before enrolment. The trial was conducted in accordance with the Declaration of Helsinki.

### Randomisation and masking

Participants were randomised to the experimental arm (procalcitonin-guided antibiotic therapy) or control arm (standard-of-care) using a computerised binary sequence number generator in a 1:1 ratio, in blocks of 14, generated by a statistician. The treatment allocations were kept in sealed opaque envelopes. Site investigators and participants were not blinded to the treatment allocation. However, all trial staff involved in data analysis were blinded to the results until completion of the statistical analysis.

### Procedures

Participants allocated to the experimental arm (PCT-guided antibiotic therapy) had serum PCT concentrations measured at enrolment and then daily until discharge, two days after cessation of antibiotics, or two days following the issuance of a non-binding advice to discontinue antibiotics, whichever occurred first. Treating physicians were advised to discontinue antibiotics: (1) at 72 h if PCT fell below 0.5 ng/mL, regardless of the baseline concentration, or, for participants with baseline PCT >2 ng/mL, if PCT decreased by ≥80% from the peak concentration; or (2) at 48 h if three consecutive PCT measurements, including the enrolment measurement, were <0.5 ng/mL, in accordance with the Asia–Pacific expert consensus supporting procalcitonin guided antibiotic de-escalation.^16^ Treating physicians were provided daily reports on participant's laboratory results including PCT levels, vital signs, and microbiological culture findings along with a printed, non-binding advice letter to support clinical decision-making on antibiotic therapy. The final decision on antibiotic therapy remained at the discretion of the treating physicians and deviations from the PCT-based non-binding advice were documented. Participants in the control arm did not have PCT concentrations measured, to avoid influencing clinician behaviour in the standard-of-care group, and antibiotics were stopped according to local guidelines or at the discretion of the treating physicians. The treating clinical team and the study team were separate, and all decisions to start or stop antibiotics were made independently by the treating team. All participants were assessed daily until discharge and at day 28 post-discharge to record the duration of antibiotic therapy, vital signs, and clinical complications. PCT concentrations were quantified using a point-of-care IMMU F6 Analyser (MedCaptain, China). Additional investigations at baseline included a complete blood count, erythrocyte sedimentation rate (ESR), serum creatinine, bilirubin and glucose. Blood cultures were collected from all participants at the moment of enrolment; additional specimens for culture were collected as clinically indicated. Microbiological culturing and antibiotic susceptibility testing were performed at Apollo Imperial Hospital Microbiology Laboratory in Chattogram following CLSI 2023 guidelines. Microbiological specimens were processed according to the laboratory SOP, including predefined specimen quality, rejection, and contamination criteria for urine, sputum, blood, and other clinical specimens. Blood culture isolates were classified as clinically significant or probable contaminants according to the laboratory SOP; only clinically significant isolates were included in the microbiological results.

Before the start of recruitment, dissemination meetings were conducted at the trial site with faculty members and trainee doctors to discuss the safety of shorter antibiotic courses, findings from previous trials, the planned study, and relevant study procedures. Patients and members of the public were not involved in the design, conduct, or reporting of the trial.

### Outcomes

The primary outcome was the length of antibiotic treatment (LOT), defined as the total number of days antibiotics were administered during the study period, up to 28 days post-hospital discharge. Participants with recurrent infections were censored at the time of diagnosis of the recurrent infection and the LOT, DOT, and DDD for the index infection were calculated up to the diagnosis of recurrent infection. For participants who left hospital, were transferred to another hospital, or did not complete day-28 follow-up, available outcomes related to antibiotic usage were used up to the last observed time point.

Secondary outcomes included length of hospital stay and antibiotic consumption during the study period up to 28 days post-hospital discharge, measured as defined daily dose (DDD), and duration of antibiotic therapy, measured as days of therapy (DOT), assessed both during hospitalisation and over the study period up to 28 days post-hospital discharge. DDD represents the assumed average antibiotic treatment dose per day for that indication.^17^ DOT represents the cumulative days of antibiotic therapy, adding up the duration of each component of antibiotic therapy if more than one antibiotic was administered. Safety outcomes included overall case fatality, mortality associated with recurrent infections, and non-lethal recurrent infections. Recurrent infections were defined as the recurrence of symptoms after cessation of antibiotics and within 28 days post-discharge. Antimicrobial susceptibility was assessed in all positive bacterial cultures using CLSI criteria. Presence of extended spectrum beta-lactamase producing bacteria was assessed by a dual disk method. A cost analysis was conducted to compare the direct costs of prescribed antibiotics and PCT assays between the two study arms. Adverse events and serious adverse events were assessed and graded according to the Common Terminology Criteria for Adverse Events (CTCAE), version 5.0.

### Statistical analysis

The sample size of the study was based on an expected reduction of two days in the mean length of antibiotic treatment (LOT) in sepsis patients receiving procalcitonin-guided antibiotic therapy, compared to a mean LOT in the control arm of 5.2 days (SD = 7.5 days), estimated from retrospective unpublished data from CMCH. With a significance level of 5% and a power of 80%, this translated into 253 participants in each study arm, which was increased to 266 participants per study arm to allow for a 5% loss to follow-up or withdrawal.

The primary analysis followed the intention-to-treat principle using an available-case approach, with no imputation of missing data. For participants who left hospital, were transferred to another hospital, or did not complete day-28 follow-up, antibiotic-use outcomes were calculated using data available up to the last observed time point; these participants were included in the intention-to-treat analysis but excluded from the per-protocol analysis. One participant in the PCT-guided arm was found to be ineligible immediately after randomisation, received no allocated intervention, and had no primary outcome data; this participant was therefore not included in the primary outcome analysis. The primary analysis was complemented by a per-protocol analysis. For participants subsequently found to have a clear alternative diagnosis, empirical antibiotic therapy was included in the primary outcome up to the point at which the alternative diagnosis was established; thereafter, antibiotic use was censored for the primary outcome, as specified in the Statistical Analysis Plan. The per-protocol analysis excluded participants who received an alternative diagnosis, left hospital against medical advice, were transferred to another hospital, or did not complete day-28 follow-up. The primary outcome, length of antibiotic therapy, and other continuous outcomes were compared between study arms using Wilcoxon's rank-sum test or an unpaired two-sample t-test, where appropriate. A Statistical Analysis Plan (SAP) was finalised and uploaded on the trial registry before database lock. All analyses were performed using “R” software (version 4.2.1). An independent Data Safety and Monitoring Board (DSMB), who were not involved in the trial design, recruitment or completion, had full access to the study data and performed an interim safety analysis after 40 and 250 participants were recruited. The trial is registered with ClinicalTrials.gov (NCT05955612).

### Role of the funding source

The study was funded by the Wellcome Trust of Great Britain (grant number 220211/A/20/Z), who was not involved in the study design, data collection, data analysis, data interpretation, or the writing of the report.

## Results

**Fig. 1:**
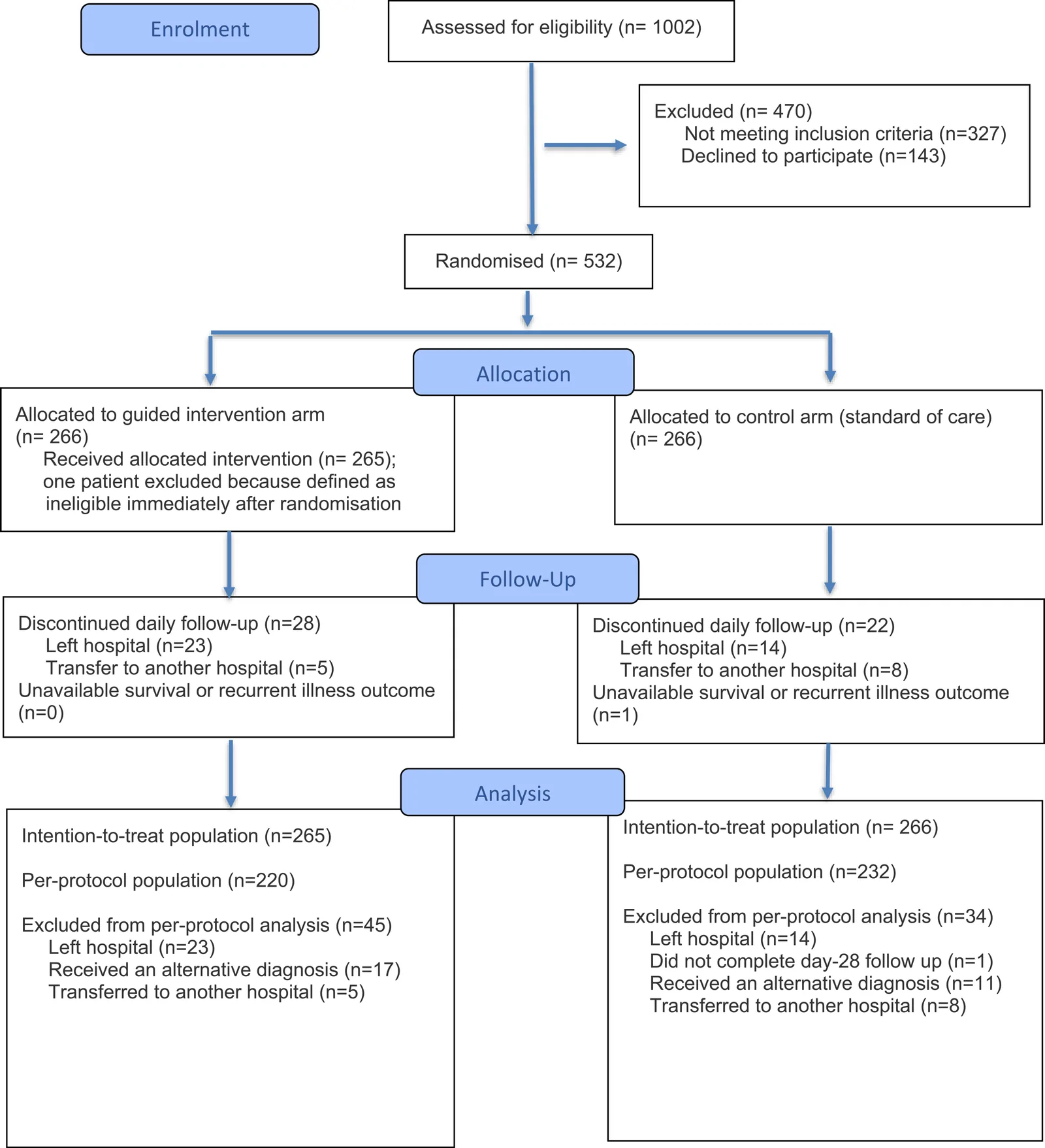
CONSORT 2025 flow diagram.

Participants were recruited between 26 July 2023 and 20 June 2024, and follow-up was completed on 30 July 2024. A total of 1002 patients were screened for eligibility, of whom 532 participants were randomised, with 266 allocated to each study arm. Primary outcome data were available for 265 participants in the PCT-guided arm and 266 participants in the control arm. One participant in the PCT-guided arm was found to be ineligible immediately after randomisation, received no allocated intervention, and had no primary outcome data. One participant in the control arm did not complete day-28 follow-up but had antibiotic data available up to hospital discharge; this participant was included in the intention-to-treat analysis and excluded from the per-protocol analysis, as prespecified. The per-protocol population comprised 452 of the 532 randomised participants; 80 were not included, comprising one participant found ineligible immediately after randomisation and 79 participants excluded according to the prespecified per-protocol criteria (Fig. 1). Baseline characteristics were well balanced between study arms (Table 1). Median patient age was 45 years (IQR 30–55), and the median NEWS score was 9 (IQR 8–10).

**Table 1:** Baseline characteristics of study participants.

Variable	Procalcitonin arm (n = 265)^a^	Control arm (n = 266)	Total (n = 531)
Age (years)	45 (32–55)	45 (28–56)	45 (30–55)
Female n (%)	133 (50%)	127 (48%)	260 (49%)
BMI (kg/m^2^)	23.1 (20.2–26.0)	22.4 (19.9–25.1)	22.8 (20.1–25.7)
Temperature (°C)	38.3 (37.8–38.9)	38.3 (37.3–38.9)	38.3 (37.6–38.9)
Respiratory rate (breaths/min)	26 (24–30)	26 (24–30)	26 (24–30)
Heart rate (beats/min)	110 (102–118)	112 (102–118)	111 (102–118)
Systolic blood pressure (mmHg)	107 (100–119)	108 (100–118)	107 (100–118)
Diastolic blood pressure (mmHg)	70 (62–77)	70 (62–78)	70 (62–78)
SpO2	96 (94–98)	96 (94–98)	96 (94–98)
Haemoglobin, g/dL	11.6 (10.2–12.7)	11.6 (10.0–13.1)	11.6 (10.1–12.9)
WBC (Total/mm^3^)	12,500 (9000–17,200)	12,700 (9500–16,650)	12,600 (9000–16,800)
Serum bilirubin, mg/dL	0.6 (0.5–0.9)	0.6 (0.5–1.0)	0.6 (0.5–1.0)
Serum creatinine, mg/dL	0.9 (0.7–1.2)	0.9 (0.7–1.1)	0.9 (0.7–1.1)
NEWS score	9 (8–10)	9 (8–10)	9 (8–10)
Modified SOFA score^b^	1 (0–3)	1 (0–2)	1 (0–2)
Initial infection diagnosis n (%)			
Enteric Fever	26 (9.8)	42 (15.8)	68 (12.8)
Intra-abdominal Infection	0 (0)	3 (1.1)	3 (0.6)
Lower respiratory tract infection	118 (44.5)	133 (50)	251 (47.3)
Meningoencephalitis	4 (1.5)	5 (1.9)	9 (1.7)
Skin and soft tissue infection	3 (1.1)	3 (1.1)	6 (1.1)
Undifferentiated sepsis	37 (13.9)	28 (10.5)	65 (12.2)
Upper respiratory tract infection	3 (1.1)	1 (0.4)	4 (0.8)
Urinary tract infection	74 (27.9)	51 (19.2)	125 (23.5)
Baseline complications/organ dysfunction n (%)			
Pulmonary oedema	4 (1.5)	9 (3.4)	13 (2.4)
Renal failure	37 (14.0)	28 (10.5)	65 (12.2)
Presence of shock	10 (3.8)	18 (6.8)	28 (5.3)
Fluid bolus required	22 (8.3)	26 (9.8)	48 (9.0)
On inotropes	4 (1.5)	10 (3.8)	14 (2.6)
Comorbidities n (%)			
Any comorbidity	122 (46)	127 (47)	249 (46)
Diabetes mellitus	73 (27.5)	73 (27.4)	146 (27.4)
Cardiac/vascular disease	70 (26.4)	67 (25.2)	137 (25.8)
Hypertension	61 (23.0)	54 (20.3)	115 (21.6)
Ischaemic heart disease	21 (7.9)	26 (9.8)	47 (8.8)
Heart failure	6 (2.3)	3 (1.1)	9 (1.7)
Chronic kidney disease	6 (2.3)	6 (2.3)	12 (2.3)
COPD	25 (9.4)	31 (11.7)	56 (10.5)
Bronchial asthma	12 (4.5)	15 (5.6)	27 (5.1)
Bronchiectasis	1 (0.4)	3 (1.1)	4 (0.8)
Neoplasm, past and/or current	0 (0.0)	1 (0.4)	1 (0.2)

BMI = body-mass index; COPD = chronic obstructive pulmonary disease; FiO_2_ = fraction of inspired oxygen; IQR = interquartile range; NEWS = National Early Warning Score; PCT = procalcitonin; SOFA = Sequential Organ Failure Assessment; SpO_2_ = peripheral oxygen saturation; WBC = white blood cell count.

a One participant in the PCT arm was found ineligible immediately after randomisation.

b Respiratory SOFA scores were based on the SpO_2_/FiO_2_ ratio; component-wise scores are provided in Tables S7–S10.

In the ITT analysis median LOT in patients receiving PCT-guided therapy was 4.7 days (IQR 2.8–8.0), compared to 9.0 days (IQR 5.8–12.0) in the control arm receiving standard-of-care (p < 0.001; Table 2). In the per protocol analysis, the median LOT was 5.0 days (IQR 3.0–8.8) in the PCT arm and 9.0 days (IQR 6.8–12.7) in the control arm (p < 0.001, Table S1).

**Table 2:** Study outcomes.

Outcome	Variable	Procalcitonin (N = 265)^a^	Control (N = 266)^b^	p-value
Primary	Length of antibiotic therapy (LOT) in days (median, IQR)	4.7 (2.8–8.0)	9.0 (5.8–12.0)	<0.001
Secondary	Days of therapy (DOT) (median, IQR)	7.2 (3.9–12.3)	13.2 (7.8–19.8)	<0.001
	Defined Daily Dose (DDD) (median, IQR)	12.0 (6.1–21.0)	21.0 (12.7–30.3)	<0.001
	Length of hospital stay in days (median, IQR)	4.7 (3.2–6.4)	4.0 (2.8–5.9)	0.003
	Length of intravenous antibiotic therapy in days (median, IQR)	3.7 (2.7–6.0)	4.7 (2.8–7.8)	0.011
	Mortality n (%)	23 (8.7)	21 (7.9)	0.875
	Recurrent infections n (%)	8 (3.0)	10 (3.8)	0.811
	Cost analysis			
	Costs of antibiotics (USD)	$37.1 (21.54–80.1)	$45.8 (30.4–83.6)	0.009
	Costs of PCT testing per patient (USD)	$16	$0	NA
	Costs of antibiotic and PCT testing combined (USD)	$53.1 (37.6–96.1)	$45.8 (30.4–83.6)	0.001

IQR = interquartile range; NA = not applicable; PCT = procalcitonin; USD=US dollars.

a One participant in the PCT arm was found ineligible immediately after randomisation.

b The denominator for mortality and recurrent infection is 265 in the control arm because one participant did not complete day-28 follow-up; these outcomes were analysed using available cases.

Table 2 shows the reduction in additional measures of antibiotic usage in patients receiving PCT-guided therapy compared to conventional treatment, including DOT (median 7.2 days, compared to 13.2 days) and DDD for individual patients (12.0 compared to 21.0). The median length of hospital stay (LOS) was 4.7 days (IQR 3.2–6.4) in the PCT-guided arm compared to 4.0 days (IQR 2.8–5.9) in the control arm (p = 0.003; Fig. S1). After 28 days, case fatality was 23/265 (8.7%; 95% CI, 5.6%–12.7%) in the procalcitonin-guided arm and 21/265 (7.9%; 95% CI, 5%–11.9%) in the control arm. The absolute risk difference was 0.8 percentage points (95% CI, −3.9 to 5.5; p = 0.875). All fatal and ICU referral cases were reviewed by the DSMB, and none were identified as related to the discontinuation of antibiotic therapy. Recurrent infection was identified in 8/265 (3.0%) participants in the PCT-guided arm, compared to 10/265 (3.8%) participants in the control group (p = 0.811). The per-protocol population analysis provided similar results as the ITT analysis (Table S1).

A total of 98/531 (18.4%) participants had a positive bacterial culture of any specimen obtained at enrolment. Blood cultures were positive in 18/265 (6.8%) participants in the PCT-guided patient group and 17/266 (6.4%) participants in the control group (Table S2). In addition, 46 participants had a positive sputum culture, and 37 participants had a positive urine culture (Table S3). *E coli* was the most frequently isolated organism (n = 41), followed by *K pneumoniae* (n = 28). Out of 41 *E coli* isolates, 30 isolates showed extended-spectrum beta-lactamase (ESBL) production assessed by a dual disk method, and 3 isolates were considered carbapenem-resistant enterobacteriaceae (CRE) (Fig. S2, Table S4).

In a post-hoc exploratory analysis, we examined patients in the PCT-guided arm with blood culture positive for ESBL-producing E coli who initially received empirical treatment with a third-generation cephalosporin. Out of 8 patients in the PCT-guided treatment arm with a blood culture positive for ESBL-producing *E coli* and receiving initial empirical antibiotic therapy with a third-generation cephalosporin, 6 patients showed rapid decline of serum PCT concentrations during the first three days of antibiotic therapy (Figs. S2 and S3), and all patients showed early clinical improvement. After susceptibility results became available, antibiotic therapy in these patients was escalated after 3–5 days informed by the antibiogram (Fig. S4). Antibiotic susceptibility profiles of all bacterial isolates obtained in the study are summarised in Fig. S5.

The median cost of antibiotic therapy was 37.1 USD (IQR 21.54–80.1) in the PCT-guided arm compared to 45.8 USD (IQR 30.41–83.6) in the standard-of-care group (p = 0.009). The median number of PCT tests utilised in the intervention arm was 4 (IQR 3–6), costing 4 USD per test. Combining the costs of antibiotics and PCT testing, the median total cost per patient was 53.1 USD (IQR 37.5–96.1) in the PCT arm compared to 45.8 USD (IQR 30.4–83.6) in the control group (Table 2). Adverse events and serious adverse events are reported as secondary outcomes and in Table 3 and Table S5 (Supplementary Material). According to a detailed review by the Data Safety Monitoring Board, no adverse events were considered related to the study intervention.

**Table 3:** Adverse events.

Adverse events	Procalcitonin arm (N = 265)^a^		Control arm (N = 266)		Total (N = 531)
Number of adverse events according to grade	1–2	3–5	1–2	3–5	7
	4	2	1	0	
Adverse event category, N (%)					
Septic thrombophlebitis	1 (0.4)	0	0	0	1 (0.2)
Skin reaction	1 (0.4)	0	0	0	1 (0.2)
Diarrhoea	0	1 (0.4)	0	0	1 (0.2)
Allergic reaction	1 (0.4)	0	1 (0.4)	0	2 (0.4)
Blood transfusion reaction	1 (0.4)	0	0	0	1 (0.2)
Fungal Pneumonia	0	1 (0.4)	0	0	1 (0.2)

a One participant in the PCT arm was found ineligible immediately after randomisation.

Among the 265 patients receiving PCT-guided therapy, their treating physicians received non-binding advice (NBA) to discontinue antibiotic therapy for 206/265 (77.7%) patients (Fig. S6). The advice to discontinue antibiotics was followed for 109/206 (53.0%) patients whereas in 97/206 (47.0%) patients antibiotic therapy continued. The primary reason for non-adherence with the NBA was persisting severity of illness, reported in 66/97 (68.0%) of participants (Table S6). In the 66 patients with persisting severity of illness, 21 had a positive bacterial culture result, of which 12 showed resistance to the initial antibiotic therapy according to the antibiogram. The alternative diagnoses of the 28 participants who were initially diagnosed with suspected sepsis and commenced on empiric intravenous antibiotic therapy, but were subsequently diagnosed to have an alternative diagnosis, are presented in Table S11.

## Discussion

In this single-centre trial in Bangladesh, procalcitonin-guided de-escalation almost halved the median duration of antibiotic therapy in adults with clinically suspected bacterial sepsis. Mortality and recurrent infections were similar between the two groups.

The reduction in the duration of antibiotic therapy observed in our study suggests that an affordable point-of-care PCT test could be an additional tool to reduce antibiotic drug pressure in hospitals in LMICs and may contribute to efforts to mitigate antimicrobial resistance. Our findings align with evidence from high-income countries. The PRORATA, SAPS, and ADAPT-Sepsis studies all showed that procalcitonin-guided protocols safely shorten antibiotic treatment duration in patients with critical illness.^11^^,^^12^^,^^18^ Unlike these studies, the current study in a low-resource setting was implemented in both the ICU and general wards, evaluating the performance of this strategy in a setting with restricted diagnostic facilities and limited provision of ICU care. Antibiotic usage was quantified by different measures,^19^ including LOT, DOT and DDD, all providing comparable results in both the intention-to-treat and the per-protocol analyses. We also observed that the procalcitonin-guided strategy reduced the duration of intravenous antibiotic therapy.

The observed numbers of deaths and recurrent infections were similar between the groups, and the Data Safety Monitoring Board did not identify any deaths as related to antibiotic discontinuation. The 95% CI around the mortality difference was compatible with both a small reduction and a modest increase in mortality. Therefore, there was no evidence of a difference in mortality or recurrent infections between the two arms. Length of hospital admission was half a day longer in the intervention arm compared to the control group. This might have been driven by awareness of the treating physicians of study arm allocation in this unblinded study, resulting in a more cautious approach to discharge patients in the PCT intervention arm receiving a shorter course of antibiotic therapy. A meta-analysis including 16 RCTs involving 6452 critically ill patients in HICs, found that procalcitonin-guided antibiotic therapy did not affect hospital length of stay compared with standard care.^20^

Other biomarkers than PCT have also been evaluated for their potential use to guide de-escalation of antibiotic therapy, including CRP.^21^^,^^22^ ADAPT-Sepsis included both PCT-guided and CRP-guided arms and showed that PCT-guided care safely reduced antibiotic duration compared with standard care, whereas CRP-guided care did not show the same reduction.^18^ A 2023 meta-analysis of three randomised trials (n = 727) found that CRP-guided protocols reduced antibiotic duration by 1.8 days without increasing mortality, compared to a reduction of 4.3 days in our study using PCT guidance.^23^

Adherence of the treating physicians to the non-binding advice to discontinue antibiotic treatment within 24 h was 53%. Adherence to the non-binding advice should be interpreted in the context of previous PCT-guided trials, where biomarker-based stopping recommendations were also advisory and could be overruled by treating clinicians when clinical concerns persisted.^11^^,^^12^ The most frequent reported reason for non-adherence was persistent clinical severity. Although persistent clinical severity was the most common recorded reason for continuing antibiotics despite PCT advice, not all cases were supported by microbiological confirmation or documented resistance, and decisions reflected the treating clinician's overall clinical assessment. This highlights that discontinuation of antibiotic therapy should not be based solely on the serum PCT concentration dynamics but should be corroborated by a clinical assessment of the patient.

Costs of deploying a PCT-based strategy for antibiotic de-escalation are an important aspect for future deployment of this strategy in LMICs. In Bangladesh, out-of-pocket payments constitute approximately 67% of total health expenditure.^24^ By reducing the duration of antibiotic therapy, PCT-guided antibiotic de-escalation decreases expenditure on antibiotics. The added cost of PCT testing resulted in a modest increase in overall direct treatment costs per patient, but this finding should be interpreted in the context of the higher costs associated with managing drug-resistant infections.^25^^,^^26^ For instance, an increase in ESBL-producing gram negative infections will necessitate a shift away from 3rd-generation cephalosporins as initial empirical treatment in severe infections towards more expensive antibiotics such as meropenem.^27^ Drug-resistant infections increase healthcare costs; for example, in Ghana, antibiotic-resistant bloodstream infections were associated with 5–8 additional hospital days and approximately 1300 USD in extra costs per patient, alongside substantial productivity losses.^28^ This cost analysis was limited to direct costs of prescribed antibiotics and PCT testing. Hospital stay costs were not included because patients are not charged a standard daily inpatient bed fee in the government hospital setting, and a reliable standard inpatient cost estimate was not available for this analysis. A more detailed health economic evaluation, including broader health-system costs and AMR-related costing, is planned separately.

In a small post-hoc exploratory subgroup of patients with ESBL-producing *E coli* bacteraemia, some patients receiving initial intravenous third-generation cephalosporin monotherapy showed early clinical improvement and a decline in serum PCT concentrations before treatment was adjusted according to susceptibility results. Given the small sample size, these observations should be interpreted cautiously and require further study, including assessment of third-generation cephalosporin minimum inhibitory concentrations and genotyping of the infecting bacteria.

This study has several limitations. It was a single-centre, open-label trial, which may have influenced clinician behaviour, including decisions on antibiotic discontinuation and hospital discharge. The study was not powered for mortality. Eligibility was based on clinically suspected bacterial sepsis and NEWS rather than Sepsis-3 or SOFA ≥2; modified SOFA was calculated retrospectively for descriptive purposes, and the low scores indicate relatively mild organ dysfunction. The low pathogen detection rate should be interpreted in the context of routine sepsis care in Bangladesh. Baseline PCT was measured only in the intervention arm; therefore, baseline PCT concentrations could not be directly compared between groups, and chance imbalance in this biomarker cannot be excluded. We also did not collect detailed clinician-level data on the decision to initiate empirical antibiotics before enrolment. Patients were enrolled after the treating team had already decided that parenteral antibiotics were clinically indicated.

In conclusion, this study shows that daily PCT-guided advice reduced the duration of antibiotic therapy in adults with a clinical diagnosis of bacterial sepsis in this single-centre trial. Mortality and recurrent infections were similar between groups, although larger multicentre studies in similar settings are needed to assess safety and generalisability.

## Contributors

FUHC, EdJ, CS, MN, MJU, MA, AG, KP, LP, PG, MM, and AMD designed the study and contributed to trial organisation. FUHC, RAMEU, PKD, DD, AHH, MK, SDG, FBJ, SIK, KC, MMo, AAS, MHOR, NUC, MAS, AG, KP, and LP recruited participants and collected samples. FUHC, DD, AHH, MK, SDG FBJ, SIK, and KC conducted clinical assessments, collected study data, and followed up participants. FUHC, MMo, AAS, MHOR, NUC, AG, KP, and LP trained the study teams. MAH led the microbiological analyses, and MA assisted with development of the laboratory protocols. MS led data management. MM performed the sample size calculations and statistical analyses, with study-related input from FUHC. FUHC, KP, LP, PG, MM, and AMD drafted the manuscript. MS, MM, and AMD accessed and verified the data. All authors reviewed the manuscript, had full access to all the data in the study, and had final responsibility for the decision to submit for publication.

## Data sharing statement

Deidentified individual participant data, data dictionary and statistical analysis codes underlying the results presented in this paper will be made available upon reasonable request to the MORU Data Access Committee (datasharing@tropmedres.ac).

## Declaration of interests

We declare no competing interests.

## Appendix A Supplementary data

Supplementary Figures and Tables

Checklist updated

PROCALBAN protocol

## References

[1] Mulchandani R., Tiseo K., Nandi A. (2025). Global trends in inappropriate use of antibiotics, 2000–2021: scoping review and prevalence estimates. BMJ Public Health.

[2] Dondorp A.M., Limmathurotsakul D., Ashley E.A. (2018). What's wrong in the control of antimicrobial resistance in critically ill patients from low- and middle-income countries?. Intensive Care Med.

[3] Rudd K.E., Johnson S.C., Agesa K.M. (2020). Global, regional, and national sepsis incidence and mortality, 1990–2017: analysis for the Global Burden of Disease Study. Lancet.

[4] Leung L.Y., Huang H.L., Hung K.K. (2024). Door-to-antibiotic time and mortality in patients with sepsis: systematic review and meta-analysis. Eur J Intern Med.

[5] Murray C.J.L., Ikuta K.S., Sharara F. (2022). Global burden of bacterial antimicrobial resistance in 2019: a systematic analysis. Lancet.

[6] Cox J.A., Vlieghe E., Mendelson M. (2017). Antibiotic stewardship in low- and middle-income countries: the same but different?. Clin Microbiol Infect.

[7] Pokharel S., Raut S., Adhikari B. (2019). Tackling antimicrobial resistance in low-income and middle-income countries. BMJ Glob Health.

[8] Arulappen A.L., Khan A.H., Danial M. (2025). Baseline predictors of antibiotics de-escalation from empirical therapies in an intensive care unit: a five-year retrospective study. BMC Infect Dis.

[9] Delévaux I., André M., Colombier M. (2003). Can procalcitonin measurement help in differentiating between bacterial infection and other kinds of inflammatory processes?. Ann Rheum Dis.

[10] Becker K.L., Nylén E.S., White J.C., Müller B., Snider R.H. (2004). Procalcitonin and the calcitonin gene family of peptides in inflammation, infection, and sepsis: a journey from calcitonin back to its precursors. J Clin Endocrinol Metab.

[11] De Jong E., Van Oers J.A., Beishuizen A. (2016). Efficacy and safety of procalcitonin guidance in reducing the duration of antibiotic treatment in critically ill patients: a randomised, controlled, open-label trial. Lancet Infect Dis.

[12] Bouadma L., Luyt C.E., Tubach F., PRORATA trial group (2010). Use of procalcitonin to reduce patients' exposure to antibiotics in intensive care units (PRORATA trial): a multicentre randomised controlled trial. Lancet.

[13] Evans L., Rhodes A., Alhazzani W. (2021). Surviving sepsis campaign: international guidelines for management of sepsis and septic shock 2021. Intensive Care Med.

[14] Hopewell S., Chan A.W., Collins G.S. (2025). CONSORT 2025 statement: updated guideline for reporting randomised trials. BMJ.

[15] Usman O.A., Usman A.A., Ward M.A. (2019). Comparison of SIRS, qSOFA, and NEWS for the early identification of sepsis in the Emergency Department. Am J Emerg Med.

[16] Lee C.C., Kwa A.L.H., Apisarnthanarak A. (2020). Procalcitonin (PCT)-guided antibiotic stewardship in Asia-Pacific countries: adaptation based on an expert consensus meeting. Clin Chem Lab Med.

[17] World Health Organization WHO Collaborating Centre for Drug Statistics Methodology. ATC/DDD Index 2025. https://www.whocc.no/atc_ddd_index/.

[18] Dark P., Hossain A., McAuley D.F., ADAPT-Sepsis Collaborators (2025). Biomarker-guided antibiotic duration for hospitalized patients with suspected sepsis: the ADAPT-sepsis randomized clinical trial. JAMA.

[19] Stanić Benić M., Milanič R., Monnier A.A. (2018). Metrics for quantifying antibiotic use in the hospital setting: results from a systematic review and international multidisciplinary consensus procedure. J Antimicrob Chemother.

[20] Peng F., Chang W., Xie J.F., Sun Q., Qiu H.B., Yang Y. (2019). Ineffectiveness of procalcitonin-guided antibiotic therapy in severely critically ill patients: a meta-analysis. Int J Infect Dis.

[21] Kubo K., Sakuraya M., Sugimoto H. (2024). Benefits and harms of Procalcitonin- or C-Reactive protein-guided antimicrobial discontinuation in critically ill adults with sepsis: a systematic review and network meta-analysis∗. Crit Care Med.

[22] Zhu Q., Wang H., Chen L., Yu Y., Chen M. (2025). Comparison of the accuracy of procalcitonin, neutrophil CD64, and C-reactive protein for the diagnosis and prognosis of septic patients after antibiotic therapy. Pract Lab Med.

[23] Dias R.F., De Paula A.C.R.B., Hasparyk U.G. (2023). Use of C-reactive protein to guide the antibiotic therapy in hospitalized patients: a systematic review and meta-analysis. BMC Infect Dis.

[24] WHO. Bangladesh National Health Accounts (2017). An overview on the public and private expenditures in health sector [news release]. https://www.who.int/bangladesh/news/detail/05-10-2017-bangladesh-national-health-accounts-an-overview-on-the-public-and-private-expenditures-in-health-sector.

[25] Kip M.M.A., Kusters R., Ijzerman M.J., Steuten L.M.G. (2015). A PCT algorithm for discontinuation of antibiotic therapy is a cost-effective way to reduce antibiotic exposure in adult intensive care patients with sepsis. J Med Econ.

[26] Garnfeldt V.M., Vincent J.L., Gruson D., Gu W.J. (2023). The budget impact of procalcitonin-guided antibiotic stewardship compared to standard of care for patients with suspected sepsis admitted to the intensive care unit in Belgium. PLoS One.

[27] Tan S.Y., Khan R.A., Khalid K.E., Chong C.W., Bakhtiar A. (2022). Correlation between antibiotic consumption and the occurrence of multidrug-resistant organisms in a Malaysian tertiary hospital: a 3-year observational study. Sci Rep.

[28] Otieku E., Fenny A.P., Labi A.K., Ofori A.O., Kurtzhals J.A.L., Enemark U. (2023). Attributable patient cost of antimicrobial resistance: a prospective parallel cohort study in two public teaching hospitals in Ghana. Pharmacoecon Open.

