# Supplementary material for: Use of procalcitonin point-of-care testing to guide de-escalation of antibiotic therapy in adults with suspected bacterial sepsis in a tertiary hospital in Bangladesh: a randomised controlled open-label trial
Source: eClinicalMedicine. 2026 Sep 2;99:104171. doi: 10.1016/j.eclinm.2026.104171 (PMC13559825; doi:10.1016/j.eclinm.2026.104171)
Supplement: Supplementary file 1 [file mmc1.docx]

**Table of contents**

[Figure S 1. Length of hospital stay in the procalcitonin-guided and control	2](#_Toc232107396)

[Figure S 2.Procalcitonin dynamics in multidrug-resistant E coli bacteraemia, N=10	3](#_Toc232107397)

[Figure S 3. Procalcitonin dynamics of individual patients with multidrug-resistant E. coli bacteraemia	3](#_Toc232107398)

[Figure S 4. Antibiotic course of individual patients with multidrug-resistant E coli bacteraemia	4](#_Toc232107399)

[Figure S 5 Heatmap of phenotypic antimicrobial susceptibility profiles of all positive cultures	4](#_Toc232107400)

[Figure S 6. Adherence of non-binding advice	5](#_Toc232107401)

[Table S 1. Per-protocol analysis findings	6](#_Toc232107385)

[Table S 2.Distribution of cultured microorganisms after deduplication (N = 98 isolates)	6](#_Toc232107386)

[Table S 3. Prevalence of culture-positive clinical sample types before deduplication (N = 120)	7](#_Toc232107387)

[Table S 4. Resistance profile of Escherichia coli	7](#_Toc232107388)

[Table S 5. Summary of severe adverse events in PCT-guided arm and standard-of-care arm	7](#_Toc232107389)

[Table S 6. Reasons for NBA non-adherence within 24 hours by participants	8](#_Toc232107390)

[Table S 7. Comparison of Total Baseline Modified SOFA Score Between Treatment Arms	9](#_Toc232107391)

[Table S 8. Descriptive Statistics for Total Modified SOFA and Component Scores	9](#_Toc232107392)

[Table S 9. Distribution of Individual SOFA Component Scores	10](#_Toc232107393)

[Table S 10. Distribution of Total Modified SOFA Score	11](#_Toc232107394)

[Table S 11. List of Alternative Diagnoses	13](#_Toc232107395)

Trial registration number: PROCALBAN (NCT05955612)


Title
Use of procalcitonin point-of-care test to guide de-escalation of empiric antibiotic therapy in adult patients with sepsis in a tertiary hospital in Bangladesh (PROCALBAN): a randomised, controlled, open-label trial **Supplementary figures**

Figure S 1. Length of hospital stay in the procalcitonin-guided and control

Figure S 2.Procalcitonin dynamics in multidrug-resistant E coli bacteraemia, N=10
CRE=carbapenem-resistant Enterobacterales; ESBL=extended-spectrum β-lactamase.

Figure S 3. Procalcitonin dynamics of individual patients with multidrug-resistant E. coli bacteraemia
CRE=carbapenem-resistant Enterobacterales; ESBL=extended-spectrum β-lactamase.

Figure S 4. Antibiotic course of individual patients with multidrug-resistant E. coli bacteraemia
CRE=carbapenem-resistant Enterobacterales; ESBL=extended-spectrum β-lactamase.

Figure S 5 Heatmap of phenotypic antimicrobial susceptibility profiles of all positive cultures

Figure S 6. Adherence of non-binding advice. NBA=non-binding advice; PCT=procalcitonin.

Reached the target level from PCT test

Total subjects in PRO arm

N=265 (100%)

Did not receive NBA

N=59/265 (22·3%)

Received NBA

N=206/265 (77·7%)

Adherent within **24** hrs: N=109/206 (53·0%)

Non-adherent within **24** hrs: N=97/206 (47·0%)

Adherent within **48** hrs: N=115/206 (55·8%)

Non-adherent within **48** hrs: N=91/206 (44·2%)

**Supplementary tables**

Table S 1. Per-protocol analysis findings

IQR=interquartile range

| Outcome | Characteristics | Procalcitonin  (N=220) | Control  (N=232) | P-value |
| --- | --- | --- | --- | --- |
| Primary | Length of antibiotic therapy (days): median (IQR) | 5.00 (3·00 – 8·77) | 9·04 (6·75 – 12·67) | <0·001 |
| Secondary | Day of therapy (DOT): median (IQR) | 7·48 (4·88 – 12·92) | 14·00 (9·27 – 21·52) | <0·001 |
|  | Defined Daily Dose (DDD): median (IQR) | 11·67 (6·05 – 19·00) | 21·50 (13·25 – 30·25) | <0·001 |
|  | Length of hospital stay (days): median (IQR) | 4·72 (3·54 – 6·06) | 4·07 (2·87 – 5·90) | 0·005 |

Table S 2.Distribution of cultured microorganisms after deduplication (N = 98 isolates)

| Species name | Total n (%) | Procalcitonin arm n (%) | Control arm n (%) | P value |
| --- | --- | --- | --- | --- |
| *Escherichia coli* | 41 (41·8) | 22 (47·8) | 19 (36·5) | 0·307 |
| *Klebsiella pneumoniae* | 28 (28·6) | 11 (23·9) | 17 (32·7) | 0·377 |
| *Pseudomonas aeruginosa* | 8 (8·2) | 5 (10·9) | 3 (5·8) | 0·469 |
| *Salmonella typhi* | 6 (6·1) | 3 (6·5) | 3 (5·8) | 1·000 |
| *Staphylococcus aureus* | 6 (6·1) | 1 (2·2) | 5 (9·6) | 0·210 |
| *Acinetobacter species* | 2 (2·0) | 0 (0·0) | 2 (3·8) | 0·497 |
| *Burkholderia cepacia* | 2 (2·0) | 0 (0·0) | 2 (3·8) | 0·497 |
| *Burkholderia pseudomallei* | 1 (1·0) | 1 (2·2) | 0 (0·0) | 0·469 |
| *Enterococcus faecalis* | 1 (1·0) | 1 (2·2) | 0 (0·0) | 0·469 |
| *Staphylococcus epidermidis* | 1 (1·0) | 1 (2·2) | 0 (0·0) | 0·469 |
| *Streptococcus pneumoniae* | 1 (1·0) | 1 (2·2) | 0 (0·0) | 0·469 |
| *Streptococcus pyogenes* | 1 (1·0) | 0 (0·0) | 1 (1·9) | 1·000 |

Note: Only clinically significant blood culture isolates are shown; isolates classified as contaminants according to the laboratory standard operating procedure (SOP) were excluded.

Table S 3. Prevalence of culture-positive clinical sample types before deduplication (N = 120)

| Culture type | Out of total | Of culture positive isolates |
| --- | --- | --- |
| Blood, n/N (%) | 35/532 (6·6) | 35/120 (29·2) |
| Urine, n/N (%) | 37/532 (6·9) | 37/120 (30·8) |
| Sputum, n/N (%) | 46/532 (8·6) | 46/120 (38·3) |
| Pus, n/N (%) | 1/532 (0·2) | 1/120 (0·8) |
| Blister Fluid, n/N (%) | 1/532 (0·2) | 1/120 (0·8) |

Table S 4. Resistance profile of Escherichia coli

| **Category** | **Overall** | **Procalcitonin arm** | **Control arm** |
| --- | --- | --- | --- |
| All isolates | 41 | 22 | 19 |
| **Sample type distribution** | | | |
| Blood | 18 | 11 | 7 |
| Urine | 17 | 8 | 9 |
| Sputum | 5 | 2 | 3 |
| Pus | 1 | 1 | 0 |
| **Resistance phenotype^*^** | | | |
| ESBL | 30 | 18 | 12 |
| Non-ESBL | 8 | 2 | 6 |
| CRE | 3 | 2 | 1 |

Resistance phenotype

- ESBL *E. coli*: *E. coli* resistant to third-generation cephalosporins due to extended-spectrum beta-lactamase production
- Non-ESBL *E. coli*: *E. coli* susceptible to third-generation cephalosporins
- CRE *E. coli*: *E. coli* resistant to one or more carbapenem antibiotics\
- Presence of extended spectrum beta-lactamase producing bacteria was assessed by a dual disk method

Table S 5. Summary of severe adverse events in PCT-guided arm and standard-of-care arm

| **Severe adverse events** | **Procalcitonin (N=265)** | | **Control (N=266)** | | **Total (N=531)** |
| --- | --- | --- | --- | --- | --- |
| Number of participants | 26 | | 24 | | 50 |
| Total number of events | 37 | | 36 | | 73 |
| Grading of adverse events | 1-2 | 3-5 | 1-2 | 3-5 | 73 |
|  | 0 | 37 | 0 | 36 |  |
| **Events, n/N (%)** |  |  |  |  |  |
| Deaths and ICU referral related to the study intervention such as death directly related to the early stopping of antibiotics or complications of prolong use of antibiotics | 0 | 0 | 0 | 0 | 0 |

ICU=intensive care unit; PCT=procalcitonin

Table S 6. Reasons for NBA non-adherence within 24 hours by participants

| **Reasons for NBA non-adherence within 24 hours by participants** | **Number** | **Percent** |
| --- | --- | --- |
| Patient’s illness severity | 66 | 68·04 |
| Senior clinician decided not to stop antibiotic | 13 | 13·40 |
| Antibiotic duration recommended in the CMC/STG guidelines | 6 | 6·19 |
| Decision deferred to next clinical round (by treating physician) | 2 | 2·06 |
| Other | 9 | 9·28 |
| Missing | 1 | 1·03 |
| Total | 97 | 100 |

NBA=non-binding advice

PROCALBAN baseline modified SOFA score

Table S 7. Comparison of Total Baseline Modified SOFA Score Between Treatment Arms

| Treatment arm | Total N | Available N | Mean (SD) | Median (IQR) | Range | P value |
| --- | --- | --- | --- | --- | --- | --- |
| Procalcitonin arm | 266 | 265 | 1.6 (2.1) | 1 (0, 3) | 0–13 | 0.790 |
| Control arm | 266 | 266 | 1.6 (2.2) | 1 (0, 2) | 0–15 |  |

P value is from the Wilcoxon rank-sum test comparing the procalcitonin arm and control arm. IQR=interquartile range; SD=standard deviation; SOFA=Sequential Organ Failure Assessment.

Table S 8. Descriptive Statistics for Total Modified SOFA and Component Scores

| Variable | Group | Mean | SD | Median | Q1 | Q3 | Min | Max | P value |
| --- | --- | --- | --- | --- | --- | --- | --- | --- | --- |
| Total modified SOFA score | Overall | 1.6 | 2.2 | 1 | 0 | 2 | 0 | 15 | 0.790 |
|  | Procalcitonin arm | 1.6 | 2.1 | 1 | 0 | 3 | 0 | 13 |  |
|  | Control arm | 1.6 | 2.2 | 1 | 0 | 2 | 0 | 15 |  |
| Respiratory component | Overall | 0.4 | 0.9 | 0 | 0 | 0 | 0 | 4 | 0.330 |
|  | Procalcitonin arm | 0.4 | 0.9 | 0 | 0 | 0 | 0 | 4 |  |
|  | Control arm | 0.4 | 0.9 | 0 | 0 | 0 | 0 | 4 |  |
| Coagulation component | Overall | 0.2 | 0.6 | 0 | 0 | 0 | 0 | 4 | 0.464 |
|  | Procalcitonin arm | 0.2 | 0.7 | 0 | 0 | 0 | 0 | 4 |  |
|  | Control arm | 0.2 | 0.6 | 0 | 0 | 0 | 0 | 4 |  |
| Liver component | Overall | 0.3 | 0.6 | 0 | 0 | 0 | 0 | 4 | 0.640 |
|  | Procalcitonin arm | 0.3 | 0.6 | 0 | 0 | 0 | 0 | 3 |  |
|  | Control arm | 0.2 | 0.6 | 0 | 0 | 0 | 0 | 4 |  |
| Cardiovascular component | Overall | 0.2 | 0.6 | 0 | 0 | 0 | 0 | 4 | 0.096 |
|  | Procalcitonin arm | 0.1 | 0.5 | 0 | 0 | 0 | 0 | 4 |  |
|  | Control arm | 0.2 | 0.7 | 0 | 0 | 0 | 0 | 4 |  |
| CNS component | Overall | 0.1 | 0.5 | 0 | 0 | 0 | 0 | 3 | 0.371 |
|  | Procalcitonin arm | 0.1 | 0.5 | 0 | 0 | 0 | 0 | 3 |  |
|  | Control arm | 0.1 | 0.5 | 0 | 0 | 0 | 0 | 3 |  |
| Renal component | Overall | 0.4 | 0.9 | 0 | 0 | 0 | 0 | 4 | 0.125 |
|  | Procalcitonin arm | 0.5 | 1.0 | 0 | 0 | 1 | 0 | 4 |  |
|  | Control arm | 0.4 | 0.9 | 0 | 0 | 0 | 0 | 4 |  |

P values in Table 2 are from Wilcoxon rank-sum tests comparing the procalcitonin arm and control arm. CNS=central nervous system; Q1=first quartile; Q3=third quartile; SD=standard deviation; SOFA=Sequential Organ Failure Assessment.

Table S 9. Distribution of Individual SOFA Component Scores

| Component | score | Overall | Procalcitonin arm | Control arm | Distribution P value |
| --- | --- | --- | --- | --- | --- |
| Respiratory component | 0 | 415 (78%) | 213 (80.1%) | 202 (75.9%) | 0.247 |
|  | 1 | 35 (6.6%) | 12 (4.5%) | 23 (8.6%) | 0.247 |
|  | 2 | 54 (10.2%) | 24 (9%) | 30 (11.3%) | 0.247 |
|  | 3 | 20 (3.8%) | 12 (4.5%) | 8 (3%) | 0.247 |
|  | 4 | 7 (1.3%) | 4 (1.5%) | 3 (1.1%) | 0.247 |
|  | Missing | 1 (0.2%) | 1 (0.4%) | 0 (0%) | 0.247 |
| Coagulation component | 0 | 464 (87.2%) | 229 (86.1%) | 235 (88.3%) | 0.748 |
|  | 1 | 41 (7.7%) | 20 (7.5%) | 21 (7.9%) | 0.748 |
|  | 2 | 14 (2.6%) | 9 (3.4%) | 5 (1.9%) | 0.748 |
|  | 3 | 6 (1.1%) | 3 (1.1%) | 3 (1.1%) | 0.748 |
|  | 4 | 6 (1.1%) | 4 (1.5%) | 2 (0.8%) | 0.748 |
|  | Missing | 1 (0.2%) | 1 (0.4%) | 0 (0%) | 0.748 |
| Liver component | 0 | 439 (82.5%) | 217 (81.6%) | 222 (83.5%) | 0.833 |
|  | 1 | 55 (10.3%) | 29 (10.9%) | 26 (9.8%) | 0.833 |
|  | 2 | 30 (5.6%) | 15 (5.6%) | 15 (5.6%) | 0.833 |
|  | 3 | 6 (1.1%) | 4 (1.5%) | 2 (0.8%) | 0.833 |
|  | 4 | 1 (0.2%) | 0 (0%) | 1 (0.4%) | 0.833 |
|  | Missing | 1 (0.2%) | 1 (0.4%) | 0 (0%) | 0.833 |
| Cardiovascular component | 0 | 482 (90.6%) | 246 (92.5%) | 236 (88.7%) | 0.182 |
|  | 1 | 38 (7.1%) | 16 (6%) | 22 (8.3%) | 0.182 |
|  | 2 | 0 (0%) | 0 (0%) | 0 (0%) | 0.182 |
|  | 3 | 0 (0%) | 0 (0%) | 0 (0%) | 0.182 |
|  | 4 | 11 (2.1%) | 3 (1.1%) | 8 (3%) | 0.182 |
|  | Missing | 1 (0.2%) | 1 (0.4%) | 0 (0%) | 0.182 |
| CNS component | 0 | 502 (94.4%) | 253 (95.1%) | 249 (93.6%) | 0.299 |
|  | 1 | 10 (1.9%) | 2 (0.8%) | 8 (3%) | 0.299 |
|  | 2 | 10 (1.9%) | 5 (1.9%) | 5 (1.9%) | 0.299 |
|  | 3 | 9 (1.7%) | 5 (1.9%) | 4 (1.5%) | 0.299 |
|  | 4 | 0 (0%) | 0 (0%) | 0 (0%) | 0.299 |
|  | Missing | 1 (0.2%) | 1 (0.4%) | 0 (0%) | 0.299 |
| Renal component | 0 | 400 (75.2%) | 192 (72.2%) | 208 (78.2%) | 0.604 |
|  | 1 | 68 (12.8%) | 37 (13.9%) | 31 (11.7%) | 0.604 |
|  | 2 | 35 (6.6%) | 21 (7.9%) | 14 (5.3%) | 0.604 |
|  | 3 | 13 (2.4%) | 7 (2.6%) | 6 (2.3%) | 0.604 |
|  | 4 | 15 (2.8%) | 8 (3%) | 7 (2.6%) | 0.604 |
|  | Missing | 1 (0.2%) | 1 (0.4%) | 0 (0%) | 0.604 |

Distribution p values in Table 3 are from chi-square tests where appropriate; Fisher exact tests with simulated p values were used when expected cell counts were small. CNS=central nervous system; SOFA=Sequential Organ Failure Assessment.

Table S 10. Distribution of Total Modified SOFA Score

| Modified SOFA score | Overall | Procalcitonin arm | Control arm |
| --- | --- | --- | --- |
| 0 | 219 (41.2%) | 112 (42.1%) | 107 (40.2%) |
| 1 | 111 (20.9%) | 48 (18%) | 63 (23.7%) |
| 2 | 78 (14.7%) | 38 (14.3%) | 40 (15%) |
| 3 | 45 (8.5%) | 26 (9.8%) | 19 (7.1%) |
| 4 | 29 (5.5%) | 14 (5.3%) | 15 (5.6%) |
| 5 | 15 (2.8%) | 10 (3.8%) | 5 (1.9%) |
| 6 | 13 (2.4%) | 8 (3%) | 5 (1.9%) |
| 7 | 7 (1.3%) | 3 (1.1%) | 4 (1.5%) |
| 8 | 6 (1.1%) | 2 (0.8%) | 4 (1.5%) |
| 9 | 4 (0.8%) | 3 (1.1%) | 1 (0.4%) |
| 11 | 1 (0.2%) |  | 1 (0.4%) |
| 12 | 1 (0.2%) |  | 1 (0.4%) |
| 13 | 1 (0.2%) | 1 (0.4%) |  |
| 15 | 1 (0.2%) |  | 1 (0.4%) |
| Missing | 1 (0.2%) | 1 (0.4%) |  |

Distribution p value for total modified SOFA score: 0.610.

Methodological note

Modified SOFA was calculated using six components: respiratory, coagulation, liver, cardiovascular, CNS, and renal. The respiratory component used SpO2/estimated FiO2 because arterial blood gas measurements were unavailable. FiO2 was estimated from recorded oxygen flow in L/min using predefined assumptions for room air, nasal cannula, facemask, and rebreather bag. The respiratory score was assigned using revised SpO2/FiO2 thresholds: >400 = 0, >315 to <=400 = 1, >235 to <=315 = 2, >150 to <=235 = 3, and <=150 = 4. The renal component was based on serum creatinine because urine output data were unavailable. The cardiovascular component used MAP calculated from systolic and diastolic blood pressure, combined with CRF-recorded inotrope/vasopressor categories where applicable

Table S 11. List of Alternative Diagnoses

| **Subject ID** | **Arm** | **Initial diagnosis** | **Alternative diagnosis** | **Date of enrolment** | **Date of alterative diagnosis** |
| --- | --- | --- | --- | --- | --- |
| PCT_BD2_026 | Control | Urinary Tract Infection (UTI) | Appendicular abscess | 17/08/2023 | 23/08/2023 |
| PCT_BD2_027 | Procalcitonin | Lower Respiratory Tract Infection (LRTI) | Pyrexia of Unknown Origin on anti-tuberculosis drug trial (empirical basis) | 18/08/2023 | 04/09/2023 |
| PCT_BD2_034 | Procalcitonin | Lower Respiratory Tract Infection (LRTI) | Adenocarcinoma of left lung with secondary lung infection | 23/08/2023 | 29/08/2023 |
| PCT_BD2_035 | Control | Undifferentiated Sepsis | Rheumatoid Arthritis with Iron Deficiency Anaemia | 23/08/2023 | 27/08/2023 |
| PCT_BD2_040 | Procalcitonin | Enteric Fever | Systemic Lupus Erythematosus (SLE) with Musculoskeletal involvement with history of Epilepsy | 26/08/2023 | 02/09/2023 |
| PCT_BD2_051 | Procalcitonin | Skin and Soft Tissue Infection | Systemic Lupus Erythematosus SLE with secondary antiphospholipid syndrome (APS) | 30/08/2023 | 10/09/2023 |
| PCT_BD2_072 | Control | Meningoencephalitis | Haemorrhagic stroke | 10/09/2023 | 12/09/2023 |
| PCT_BD2_092 | Control | Lower Respiratory Tract Infection (LRTI) | Suspected Carcinoma lung with metastasis in bone with secondary respiratory infection | 25/09/2023 | 28/09/2023 |
| PCT_BD2_098 | Control | Urinary Tract Infection (UTI) | Pyrexia of Unknown Origin on empiric anti-tuberculosis trial | 01/10/2023 | 09/10/2023 |
| PCT_BD2_105 | Procalcitonin | Lower Respiratory Tract Infection (LRTI) | Lung abscess | 04/10/2023 | 08/10/2023 |
| PCT_BD2_107 | Control | Enteric Fever | Enteric fever with complications: typhoid Ulcer perforation | 07/10/2023 | 14/10/2023 |
| PCT_BD2_125 | Procalcitonin | Urinary Tract Infection (UTI) | Suspected Abdominal Tuberculosis and started on anti-tuberculosis drug on empirical basis | 15/10/2023 | 28/10/2023 |
| PCT_BD2_147 | Control | Urinary Tract Infection (UTI) | Suspected Tubercular Meningitis with 6th Nerve Palsy | 02/11/2023 | 11/11/2023 |
| PCT_BD2_162 | Procalcitonin | Undifferentiated Sepsis | Sepsis with chronic subdural hematoma | 09/11/2023 | 12/11/2023 |
| PCT_BD2_164 | Procalcitonin | Urinary Tract Infection (UTI) | Melioidosis (required prolonged antibiotic therapy) | 11/11/2023 | 14/11/2023 |
| PCT_BD2_168 | Procalcitonin | Enteric Fever | Left sided Chronic Suppurative Otitis Media (CSOM) with Mastoid Abscess | 12/11/2023 | 08/12/2024 |
| PCT_BD2_173 | Procalcitonin | Lower Respiratory Tract Infection (LRTI) | Tubercular pleural effusion | 14/11/2023 | 19/11/2023 |
| PCT_BD2_283 | Procalcitonin | Lower Respiratory Tract Infection (LRTI) | Lung abscess with acromegaly | 24/01/2024 | 27/01/2024 |
| PCT_BD2_318 | Procalcitonin | Lower Respiratory Tract Infection (LRTI) | Sepsis due to Liver Abscess with chronic obstructive pulmonary disease (COPD) | 11/02/2024 | 15/02/2024 |
| PCT_BD2_340 | Procalcitonin | Urinary Tract Infection (UTI) | Appendicular abscess surgically treated followed by surgical site infection | 20/02/2024 | 22/02/2024 |
| PCT_BD2_346 | Control | Undifferentiated Sepsis | Suspected renal mass with abdminal lymph nodes | 22/02/2024 | 02/03/2024 |
| PCT_BD2_354 | Control | Lower Respiratory Tract Infection (LRTI) | Smear positive Pulominary Tuberculosis | 27/02/2024 | 28/02/2024 |
| PCT_BD2_362 | Procalcitonin | Lower Respiratory Tract Infection (LRTI) | Smear negative pulmonary TB with right side pleural effusion | 02/03/2024 | 06/03/2024 |
| PCT_BD2_376 | Control | Enteric Fever | Carcinoma stomach | 09/03/2024 | 20/03/2024 |
| PCT_BD2_404 | Procalcitonin | Lower Respiratory Tract Infection (LRTI) | Suspected tubercular empyema | 27/03/2024 | 01/04/2024 |
| PCT_BD2_442 | Procalcitonin | Meningoencephalitis | Acute cerebral infarct with haemorrhagic transformation with DM | 25/04/2024 | 28/04/2024 |
| PCT_BD2_453 | Control | Lower Respiratory Tract Infection (LRTI) | Smear negative suspected tubercular pleural effusion empirically started on anti TB drug | 30/04/2024 | 12/05/2024 |
| PCT_BD2_475 | Procalcitonin | Community Acquired Pneumonia | Right sided lung abscess | 13/05/2024 | 15/05/2024 |
