# Supplementary material for: Use of procalcitonin point-of-care testing to guide de-escalation of antibiotic therapy in adults with suspected bacterial sepsis in a tertiary hospital in Bangladesh: a randomised controlled open-label trial
Source: eClinicalMedicine. 2026 Sep 2;99:104171. doi: 10.1016/j.eclinm.2026.104171 (PMC13559825; doi:10.1016/j.eclinm.2026.104171)
Supplement: Supplementary file 3 [file mmc3.docx]

**Use of procalcitonin point-of-care test to guide de-escalation of empiric antibiotic therapy in adult patients with sepsis in a tertiary hospital in Bangladesh (PROCALBAN): a phase 3 randomised, controlled, open-label trial**

**Internal Reference Number: BAC23002**

**Short title:** Use of procalcitonin, a blood test to guide antibiotic therapy for sepsis in adults

**OxTREC Ref:** 12-23

**Date and Version No**: 31 January 2024, V3.0

| **Principal Investigator:** | Prof Arjen M Dondorp, Professor of Tropical Medicine, University of Oxford, and Deputy Director of Mahidol Oxford Tropical Medicine Research Unit (MORU) |
| --- | --- |
| **Investigators:** | 1. Forhad Uddin Hasan Chowdhury, DPhil Student   NDM, University of Oxford (Co- principal investigator)   2. Prof. Aniruddha Ghose (Site principal investigator)   Professor, Department of Medicine,  Chittagong Medical College & Hospital, Bangladesh   3. Prof Philippe Guerin   Director, IDDO  University of Oxford   4. Dr. Katherine Plewes, Clinical Investigator, MORU 5. Dr. Luigi Pisani, Clinical Investigator, MORU 6. Prof Md. Abdus Sattar   Professor & Head, Department of Medicine, Chattogram Medical College Hospital, Bangladesh   7. Prof. Dr. Constance Schultsz   Clinical Microbiologist, Department of Global Health, and Department of Medical Microbiology, Amsterdam University Medical Centres  Netherlands   8. Prof. Md. Jashim Uddin   Professor, Department of Medicine,  Chittagong Medical College & Hospital, Bangladesh   9. Professor Md. Harunur Or Roshid   Professor & Head, Department of  Anaesathesiology, and ICU,  Chattogram Medical College Hospital,  Bangladesh     10. Prof Maarten Nijsten, Surgical Intensivist, Department of Critical Care, University Medical Center Groningen 11. Dr Evelien de Jong, Infectious Diseases and Intensive Care Physician, Department of Intensive Care, Rode Kruis Ziekenhuis, Beverwijk, The Netherlands 12. Prof. Md. Akram Hossain, Senior Consultant, Microbiology, Molecular Biology and IPC, Apollo Imperial Hospitals, Chattogram, Bangladesh 13. Dr Pranay Kumar Dutta, Associate Professor, Department of Anaesathesiology and ICU, Chattogram Medical College & Hospital, Bangladesh 14. Dr. Rabiul Alam Md Erfan Uddin Assistant Professor, Department of Medicine, Chattogram Medical College & Hospital, Bangladesh 15. Dr. Md. Mohasin   Associate Professor, Department of Biochemistry and Molecular  Biology, University of Dhaka, Dhaka, Bangladesh   16. Dr. Abdullah Abu Sayeed   Assistant Professor, Department of Medicine  Chattogram Medical College   17. Dr. Naim Uddin Chy, Associate Professor, Department of Economics, University of Chittagong, Bangladesh 18. Prof. Mavuto Mukaka, Head of the Statistics, MORU, Bangkok |
| **Sponsor** | University of Oxford |
| **Funder:** | Wellcome Trust of Great Britain |
| **Principal Investigator Signature:**  **Statistician Signature:** |  |

**Conflict of interest**

The investigators declare no potential conflicts of interest.

**Confidentiality Statement**

This document contains confidential information that must not be disclosed to anyone other than the Sponsor, the Investigator Team, host organisation, and members of the Research Ethics Committee, unless authorised to do so.

**Use of procalcitonin point-of-care test to guide de-escalation of empiric antibiotic therapy in adult patients with sepsis in a tertiary hospital in Bangladesh (PROCALBAN): a phase 3 randomised, controlled, open-label trial**

**Protocol Date and Version No**: 31 January 2024, V3.0

**Protocol signature page**

The undersigned has read and understood the trial protocol detailed above and agrees to conduct the trial in compliance with the protocol.

| Prof Arjen M Dondorp |  |  |  |  | 03Jan. 24 |
| --- | --- | --- | --- | --- | --- |
| **Principal Investigator** |  | **Signature** |  |  | **Date** |

TABLE OF CONTENTS

[1.	KEY TRIAL CONTACTS	8](#_Toc137632328)

[2.	LAY SUMMARY	10](#_Toc137632329)

[3. SYNOPSIS	11](#_Toc137632331)

[4.	ABBREVIATIONS	15](#_Toc137632332)

[5.	BACKGROUND AND RATIONALE	16](#_Toc137632333)

[6.	OBJECTIVES AND OUTCOME MEASURES	18](#_Toc137632334)

[7.	TRIAL DESIGN	20](#_Toc137632335)

[8.	PARTICIPANT IDENTIFICATION	20](#_Toc137632336)

[8.1.	Trial Participants	21](#_Toc137632337)

[8.2	Inclusion criteria	21](#_Toc137632338)

[8.3	Exclusion Criteria	22](#_Toc137632339)

[8.4	Consort Diagram	23](#_Toc137632340)

[9	TRIAL PROCEDURES	23](#_Toc137632345)

[9.1	Recruitment	24](#_Toc137632346)

[9.2	Informed Consent	25](#_Toc137632347)

[9.3	Screening procedures	25](#_Toc137632348)

[9.4 Randomisation and blinding	26](#_Toc137632349)

[9.5 Clinical procedures	26](#_Toc137632350)

[9.5.1 Clinical assessment during baseline and initial procedures	26](#_Toc137632351)

[9.5.2 Clinical assessments during hospitalization	27](#_Toc137632352)

[9.5.3. Imaging	27](#_Toc137632354)

[9.5.4. Follow up visit	27](#_Toc137632355)

[9.6. Control group and intervention group procedures	28](#_Toc137632356)

[9.7 Assessment of Endpoints	30](#_Toc137632357)

[9.8	Sample Handling	31](#_Toc137632358)

[9.8.1 Sample handling for trial purposes	31](#_Toc137632359)

[9.8.1.1 Blood sampling on enrolment/baseline (H0)	32](#_Toc137632360)

[9.8.2 Sample handling for control arm	33](#_Toc137632361)

[9.9 Blood volumes and analysis summary	33](#_Toc137632362)

[9.10 Early Discontinuation/Withdrawal of Participants	33](#_Toc137632363)

[9.11	Definition of End of Trial	34](#_Toc137632364)

[10	TRIAL INTERVENTIONS	34](#_Toc137632365)

[10.1.1. Blinding of PCT assay	36](#_Toc137632367)

[10.1.2. Storage of procalcitonin kit and reagents	36](#_Toc137632368)

[10.1.3. Accountability of the Trial Intervention	36](#_Toc137632369)

[10.2	Other Treatments (non-IMPS)	36](#_Toc137632370)

[10.3	Other Interventions	37](#_Toc137632371)

[11	SAFETY REPORTING	37](#_Toc137632372)

[11.1	Assessment results outside of normal parameters as AEs and SAEs	37](#_Toc137632373)

[11.2	Procedures for Reporting Adverse Events	37](#_Toc137632374)

[11.2.1. Procedure for immediate reporting of Serious Adverse Events	38](#_Toc137632375)

[12	STATISTICS	38](#_Toc137632376)

[12.1	Statistical Analysis Plan (SAP)	38](#_Toc137632377)

[12.1.1	Length of therapy	38](#_Toc137632378)

[12.2	Description of Statistical Methods	38](#_Toc137632379)

[12.3	Sample Size Determination	38](#_Toc137632380)

[12.4	Analysis Population	39](#_Toc137632381)

[12.5	Stopping Rules	39](#_Toc137632382)

[12.6	The Level of Statistical Significance	39](#_Toc137632383)

[12.7	Procedure for Accounting for Missing, Unused, and Spurious Data.	39](#_Toc137632384)

[12.8	Procedures for Reporting any Deviation(s) from the Original Statistical Plan	39](#_Toc137632385)

[12.9	Health Economic Analysis	40](#_Toc137632386)

[13	DATA MANAGEMENT	40](#_Toc137632387)

[13.1	Source Data	40](#_Toc137632388)

[13.2	Access to Data	40](#_Toc137632389)

[13.3	Data Recording and Record Keeping	41](#_Toc137632390)

[14	QUALITY ASSURANCE PROCEDURES	41](#_Toc137632391)

[14.1	Risk assessment	41](#_Toc137632392)

[14.2	Monitoring	42](#_Toc137632393)

[14.3	Trial committees	42](#_Toc137632394)

[14.3.1 Trial management committee	42](#_Toc137632395)

[14.3.2 Trial steering committee	42](#_Toc137632396)

[14.3.3 Independent Data (Safely) Monitoring Committee	43](#_Toc137632397)

[15	PROTOCOL DEVIATIONS AND VIOLATIONS	44](#_Toc137632398)

[16	ETHICAL AND REGULATORY CONSIDERATIONS	45](#_Toc137632399)

[16.1	Declaration of Helsinki	45](#_Toc137632400)

[16.2	Guidelines for Good Clinical Practice	45](#_Toc137632401)

[16.3	Approvals	45](#_Toc137632402)

[16.4	Other Ethical Considerations	45](#_Toc137632403)

[16.5	Reporting	45](#_Toc137632404)

[16.6	Transparency in Research	45](#_Toc137632405)

[16.7	Participant Confidentiality	45](#_Toc137632406)

[16.8	Expenses and Benefits	46](#_Toc137632407)

[17	FINANCE AND INSURANCE	46](#_Toc137632408)

[17.1	Funding	46](#_Toc137632409)

[17.2	Insurance	46](#_Toc137632410)

[17.3	Contractual arrangements	46](#_Toc137632411)

[18	PUBLICATION POLICY	46](#_Toc137632412)

[19	ARCHIVING	46](#_Toc137632413)

[20	APPENDIX A: TRIAL FLOW CHART	47](#_Toc137632414)

[21	APPENDIX B: SCHEDULE OF SAMPLING AND PROCEDURES	48](#_Toc137632415)

[22. APPENDIX C: AMENDMENT HISTORY	50](#_Toc137632417)

[23.REFERENCES	53](#_Toc137632418)

# 1. KEY TRIAL CONTACTS

| **Principal Investigator** | Prof Arjen M Dondorp, Professor of Tropical Medicine,  University of Oxford  Deputy Director of Mahidol Oxford Tropical Medicine Research Unit (MORU)  Email: arjen@tropmedres.ac |
| --- | --- |
| **Sponsor** | University of Oxford |
| **Funder(s)** | Welcome Trust of Great Britain |
| **Clinical Trials Unit** | Greg Fegan, Head of Clinical Trials Support Group  Mahidol Oxford Tropical Medicine Research Unit  Faculty of Tropical Medicine Mahidol University  420/6 Rajvithi Road  Bangkok 10400, Thailand  Tel. +66(0)22036333  Fax. +66(0)23549169  [Greg@tropmedres.ac](mailto:Greg@tropmedres.ac) |
| **Statistician** | Mavuto Mukaka, Associate Professor, Clinical Trials Support Group  Mahidol Oxford Tropical Medicine Research Unit  Faculty of Tropical Medicine Mahdiol University  420/6 Rajvithi Road  Bangkok 10400, Thailand  Tel. +66(0)22036333  Fax. +66(0)23549169  [Mavuto@tropmedres.ac](mailto:Mavuto@tropmedres.ac) |
| **Committees** | **Trial management committee**  Members   1. Prof Arjen M Dondorp, Principal Investigator and Supervisor of DPhil 2. Dr Forhad Uddin Hasan Chowdhury- Co-Principal Investigator and DPhil Student 3. Prof. Aniruddha Ghose Professor of Medicine, Chattogram Medical College Hospital, Bangladesh 4. Prof. Dr. Constance Schultsz, Clinical Microbiologist, Department of Global Health, and Department of Medical Microbiology, Amsterdam University Medical Centres   Netherlands   5. Dr Katherine Plewes, Investigator and co-supervisor of DPhil 6. Dr Luigi Pisani, Investigator and co-supervisor of DPhil 7. Prof. Md. Akram Hossain, Senior Consultant, Microbiology, Molecular Biology and IPC, Apollo Imperial Hospitals 8. Dr Mavuto Mukaka, Chief Statistician and co-supervisor of DPhil 9. Atanu Bhattacharjee Statistician- Statistician 10. Chiraporn Taya - Statistician   Remark: The member may be modified in the future.  **Trial steering committee**  Members   1. Prof M A Faiz, Professor of Medicine and Former Director General of Health Services, Bangladesh 2. Professor Md. Ahmedul Kabir, Professor of Medicine and Additional Director General of Health Services, Bangladesh 3. Professor Md. Nazmul Islam, Director, Communicable Disease Control Programme (CDC), Government of Bangladesh 4. Satyajit Dhar, Professor, Department of Anaesthesiology and ICU, Chittagong Medical College 5. Professor Dr Ripon Barua, Professor and Head, Department of Microbiology, Chittagong Medical College, Chattogram 6. Dr Ariful Basher, Consultant Infectious Diseases, Infectious Diseases Hospital, Dhaka   Remark: The member may be modified in the future.  **Independent Data (Safety) Monitoring Committee**  Members   1. Prof Md Robed Amin, Professor of Medicine and Line Director, Non-Communicable Disease Control Programme, Bangladesh 2. Prof Zaed Hosaain, Professor of Medicine, Dhaka Medical College Hospital 3. Dr. Marc Henrion, Head, Statistical Support Unit, Malawi – Liverpool – Wellcome Programme 4. Dr. Gobinda Banik, Associate Professor of Medicine, Dhaka Medical College Hospital 5. Proposed members by the Bangladesh Medical Research Council (BMRC)   Remark: The member may be modified in the future. |

# 2. LAY SUMMARY

# Antimicrobial resistance (AMR) is a global problem, resulting in increased morbidity and mortality from multidrug resistant bacterial infections that are challenging to treat. Low- and middle-income countries (LMICs), like Bangladesh, are disproportionally affected by AMR. Excessive use of antibiotics both inside and outside the hospital is thought to be an important driver of AMR in Bangladesh and other LIMCs. Several studies have shown that sequential assessment of serum procalcitonin (PCT) concentrations is an effective and safe strategy to shorten the duration of antibiotic therapy in critically ill patients. In this strategy, a decrease of procalcitonin below a certain threshold prompts the clinician to stop or reduce antibiotic treatment. However, these studies were conducted in high resource settings with standard laboratory PCT testing at considerable costs. We propose to evaluate the use of sequential serum PCT measurement to guide de-escalation of antibiotic therapy in a randomised clinical trial in patients with sepsis in a large tertiary referral hospital in Chattogram, Bangladesh, using low-cost point-of-care quantitative procalcitonin measurements. Main endpoint of the trial will be the difference in antibiotic therapy duration between the study groups. If this study shows that PCT guided de-escalation of antibiotics results in a safe reduction in antibiotic usage, this will be an important strategy to reduce the antibiotic burden in hospitals in Bangladesh and comparable resource-limited settings, as well as other consequences of prolonged antibiotic therapy, including increased costs, prolonged duration of intravenous lines and duration of stay, and microbiota changes.

# 3. SYNOPSIS

| Trial Title | **Use of procalcitonin point-of-care test to guide de-escalation of empiric antibiotic therapy in adult patients with sepsis in a tertiary hospital in Bangladesh (PROCALBAN): a phase 3 randomised, controlled, open-label trial** | | |
| --- | --- | --- | --- |
| Internal ref. no. (or short title) | BAC23002 (Use of procalcitonin, a blood test to guide antibiotic therapy for sepsis in adults) | | |
| Trial registration | Clinicaltrials.gov | | |
| Sponsor | University of Oxford | | |
| Funder | Wellcome Trust of Great Britain | | |
| Clinical Phase | (Phase 3) | | |
| Trial Design | Randomised controlled, two-arm, parallel, clinical trial to assess the efficacy and safety of sequential daily procalcitonin assessments to guide de-escalation of empirical antibiotic therapy in adult patients with sepsis.  Patients will be 1:1 randomised to either:  Intervention arm: daily measurement of serum procalcitonin concentrations to guide de-escalation of antibiotics, or  Control arm: standard of practice to guide de-escalation of antibiotics without procalcitonin assessments.  Patients will be followed-up until ICU discharge and/or hospital discharge with an additional follow-up at 28 days after discharge. | | |
| Trial Participants | Male or female hospitalised patients, 16-65 years of age, with confirmed or suspected sepsis | | |
| Sample Size | 532  Intervention arm: 266  Control arm: 266 | | |
| Planned Trial Period | Total duration: 2 years | | |
| Planned Recruitment period | From 01 July 2023 to 30 June 2025 | | |
|  | **Objectives** | **Endpoints** | **Timepoint(s) of evaluation of this outcome measure (if applicable)** |
| **Primary objective** | To assess the **efficacy** of daily procalcitonin measurements to reduce the length of antibiotic treatment in hospitalized patients with sepsis in a low-resourced setting | **Length of antibiotic treatment (LOT) during period:** number of days of antibiotic treatment during the study period. | Length of treatment will be measured from the day of enrolment to completion of the study follow up (28 days after hospital discharge). The primary outcome data for participants with recurrent infections will be censored at the time of recurrent infection diagnosis.  . |
| **Secondary objectives** | 1. To assess if procalcitonin guided de-escalation reduces antibiotic **consumption** and days of therapy with antibiotics | Consumption of antibiotics expressed as the Defined Daily Dosage (DDD)  Definition of DDD: The basic definition for a defined daily dose is the assumed average maintenance dose per day for a drug used in its main indication in adults. Antibiotic use using the WHO DDD definition (<https://www.whocc.no/atc_ddd_index/>) will be determined for the most frequently prescribed Drugs in Bangladesh | DDD will be compared between study arms during the study period (day of enrolment to completion of the study follow up) |
|  |  | Days of therapy with antibiotics (DOT)  Definition of DOT: One DOT represents the administration of a single antibiotic on a given day regardless of the number of doses administered or dosage strength. If a patient receives more than one antibiotic it will be added on with DOT of the first antibiotic. (Dalton et al. 2015) | DOT will be measured from the day of enrolment to the day of stopping antibiotic(s). |
|  |  | Number of days of parenteral antibiotic during hospitalisation period | will be measured from the day of enrolment to the day of stopping of parenteral antibiotic during hospitalisation period |
|  |  | **Length of antibiotic treatment (LOT) during hospitalisation period:** number of days of antibiotic treatment during the hospitalisation period. | Length of treatment will be measured from the days of enrolment to stopping of antibiotic during hospitalisation period. |
|  | 2. To assess the safety of procalcitonin guided de-escalation of treatment | Overall mortality, mortality associated with recurrent infection.  Non-lethal recurrent infections.  Recurrent infections include culture positive relapse (failure of initial eradicative treatment for the original infecting strain), culture negative relapse (recurrence of initial symptoms after stopping of antibiotics and cultures negative), reinfection (infection with a different strain of the same species) and syndromic recurrence (recurrence of initial symptoms after stopping antibiotics with initial cultures negative and subsequent cultures either positive or negative or not done). Recurrent infections are defined as growth of one or more of the initial causative bacterial strains (i.e., same genus, species and antimicrobial susceptibility) from a second sample taken from the same infection site after stopping of antibiotic therapy, combined with clinical signs and symptoms of infection. Superinfection is defined as the isolation from the same or another site of one or more pathogens different from that identified during the first infectious episode, together with clinical signs or symptoms of infection. | Overall mortality, defined as mortality during hospital stay and by day 28 after discharge.  Mortality associated with recurrent infection, defined as recurrence of initial symptoms (independent of culture results).  Non-lethal recurrent infection, defined as recurrence of initial symptoms (independent of culture results) during the study period (up to 28 days after hospital discharge) |
|  | 3. To assess the effect of procalcitonin de-escalation therapy on length of hospital stay | Duration of hospital stay (ICU/ general ward) | Duration of hospital stay (from day of hospital admission to the day of discharge) |
|  | 4. To determine the spectrum of antimicrobial resistance among bacteria identified in clinical specimens from Bangladeshi patients hospitalized with sepsis | Bacterial isolates from blood cultures will be identified, tested for antibiotic susceptibility. In a subset of antibiotic resistant isolates, genetic correlates of resistance will be assessed. | Blood cultures will be obtained at the baseline and when there is any readmission during suspected recurrent infection |
|  | 5. To compare the costs of the procalcitonin-guided de-escalation approach with the potential cost-savings in antibiotic usage | Direct medical costs of prescribed antibiotics, the PCT assay and hospital stay cost. | During hospitalization |
| **Intervention arm** | Antibiotics will be started based on clinical suspicion of infection at the discretion of the treating physician. In the intervention arm, serum procalcitonin will be assessed at baseline and then daily until two days after stopping antibiotic, two days after providing non-binding advice or discharge, whichever comes first.  In patients with PCT level is between 0.5-2 ng/ml, the treating physician will receive **a non-binding advice** to stop antibiotics at 72 hours when the PCT level decreases below 0.5 ng/ml  In patients with an increased serum PCT concentration at baseline (> 2 ng/ml), the treating physician will receive **a non-binding advice** to stop antibiotics at 72 hours when the PCT level decreases to 80% from the peak value or a decrease below an absolute value of 0.5 ng/ml, whatever comes first.  In patient with baseline PCT is below 0.5 ng/ml only 3 PCT assays will be done and if consecutive 3 PCT levels are less than 0.5 ng/ml, the treating physician will receive **a non-binding advice** to stop antibiotics at 48 hours | | |
| **Comparator arm** | Antibiotic prescription and duration will be at the discretion of the treating physician. The treating physician will take decisions around discontinuation of antibiotic treatment based on clinical improvement, following hospital guidelines. | | |

# 4. ABBREVIATIONS

| AE | Adverse event |
| --- | --- |
| AMR | Antimicrobial resistance |
| AR | Adverse reaction |
| CI | Chief Investigator |
| CRF | Case Report Form |
| CT | Clinical Trials |
| CTA | Clinical Trials Authorisation |
| DMC/DMSC | Data Monitoring Committee / Data Monitoring and Safety Committee |
| GCP | Good Clinical Practice |
| ICF | Informed Consent Form |
| ICH | International Conference on Harmonisation |
| ICU | Intensive care unit |
| IMP | Investigational Medicinal Product |
| IRB | Independent Review Board |
| RES | Research Ethics Service |
| LMIC | Low- and middle-income countries |
| NEWS | National Early Warning Score |
| OXTREC | Oxford Tropical Research Ethics Committee |
| PI | Principal Investigator |
| PIL | Participant/ Patient Information Leaflet |
| PCT | Procalcitonin |
| REC | Research Ethics Committee |
| SAE | Serious Adverse Event |
| SAR | Serious Adverse Reaction |
| SDG | Sustainable Development Goals |
| SDV | Source Data Verification |

# 5. BACKGROUND AND RATIONALE

***Antimicrobial resistance and antibiotic consumption***

Antimicrobial resistance (AMR) is widely recognized as a major global problem. According to a recent World Bank report, 700,000 people die from drug-resistant infections each year, potentially reaching up to 10 million deaths annually (O'Neill 2016). Further, the WHO states that AMR will result in a3.8% reduction of annual GDP by the year 2050 if current trends continue (World Bank 2017). Countering the increasing problem of AMR has been recognized as an important pillar for reaching the Sustainable Development Goals (SDGs) (STOP AMR 2020, WHO 2020). Low- and middle-income countries (LMICs,) like Bangladesh, are disproportionally affected by AMR and its devastating consequences (Tan, Kang et al. 2021, Pokharel, Raut et al. 2019).

***Antibiotic consumption in Bangladesh***

Bangladesh, a lower-middle income country, has a high burden of AMR related mortality and morbidity, although there are very limited data available to quantify the problem (Ahmed, Rabbi et al. 2019). Limited antibiotic stewardship causing excessive use of antibiotics and problems with infection prevention and control (IPC) measures are recognized as important drivers of AMR in LMICs, related to scarcity of human and non-human resources, training, and surveillance (Dondorp, Limmathurotsakul et al. 2018, Llor, Bjerrum 2014). Establishing rational and limited use of antibiotics in both outpatient and hospitalized patients is more complicated in LMICs, like Bangladesh, for a variety of reasons. These include the lack of microbiology laboratory facilities, hampering the reliable differentiation of bacterial sepsis from other severe non-bacterial infections. This also impedes appropriate targeting and de-escalation of antimicrobial therapy guided by microbiological culture results that identify the pathogen and its antimicrobial susceptibility. In addition, empirical pre-hospitalization antibiotic treatment can lead to negative cultures in the presence of bacterial infection even when laboratory facilities are available. Narrowing down broad-spectrum antibiotics or stopping antibiotic treatment can thus in general not be based on microbiological culture results; this will often result in prolonged usage of broad-spectrum antibiotics that are potentially unnecessary. At the hospital level, reducing the duration of antibiotic use will reduce the antibiotic drug pressure on the bacterial populations, and could thus be important for reducing AMR. In addition, reduced antimicrobial usage would reduce costs and could also reduce the incidence of secondary infections, such as fungal infections.

***Procalcitonin as a biomarker to guide antibiotic therapy***

The use of biomarkers to differentiate between bacterial and viral causes of severe infections and to support decisions in de-escalating antibiotic therapy could be useful to reduce antibiotic usage in LMICs with limited microbiological laboratory facilities. A variety of biomarkers have been evaluated to distinguish between bacterial and viral causes of severe infections including procalcitonin. In patients with sepsis, procalcitonin was shown to distinguish a bacterial from a viral cause of infection with a specificity of 75 to 79% and a sensitivity of 77 to 88% in a high-income setting (Simon, Gauvin et al. 2004, Wacker, Prkno et al. 2013). When evaluated in LMIC settings, using a procalcitonin cut-off of 1-1.5 ng/ml, procalcitonin could distinguish bacterial infections with a specificity of 51 to 67% and a sensitivity of 80 to 91% (Tan, Kang et al. 2021, Ojuawo, Fawibe et al. 2021).

Procalcitonin is a prohormone of calcitonin and is synthesised by the C-cells of the thyroid gland (Davies 2015). However, in response to bacterial sepsis, the neuroendocrine cells in the lung, liver, kidney, adipocyte cells and intestine will produce procalcitonin ectopically and this process is mediated mainly by bacterial endotoxins and inflammatory cytokines (Dandona, Nix et al. 1994, Maruna, Nedelníková et al. 2000). The plasma half-life of procalcitonin is between 24-36 hours (Meisner et al.,2000) and serum procalcitonin concentrations have been shown to decrease rapidly after adequate and successful antibiotic treatment of patients with bacterial sepsis (Schuetz, Christ-Crain et al. 2009).

This has prompted studies to evaluate whether a decrease of serum procalcitonin below a certain threshold can guide the clinician to stop or reduce antibiotic treatment. A randomized trial in patients with sepsis in a Swiss hospital showed that this strategy resulted in a 4-day reduction in the duration of antibiotic therapy, without causing excess mortality or increased recurrence of the primary infection (Nobre, Harbarth et al. 2008). A large randomized controlled trial from the Netherlands also showed that sequential assessment of serum procalcitonin concentrations is an effective and safe strategy to shorten the duration of antibiotic therapy in critically ill patients (De Jong, Van Oers et al. 2016). In this study in 4507 critically ill adult patients, reduction in procalcitonin below 80% or more of its peak value or to 0·5 μg/L or lower prompted a non-binding advice to the clinician to stop antibiotic treatment in the intervention arm. The median duration of antibiotic use was 5 days in the intervention group compared to 7 days in the control arm. Several other randomized studies in different European countries confirmed the efficacy and safety of procalcitonin-guided de-escalation of antibiotics in bacterial sepsis (Bouadma, Luyt et al. 2010, Jensen, Hein et al. 2011).

***Procalcitonin guided antibiotic therapy in LMICs, including Bangladesh***

In contrast, there is very limited evidence that this strategy can be used to reduce antibiotic usage in LMICs, although one small study from Brazil in 81 patients suggested effective procalcitonin guided reduction in antibiotic use (Deliberato, Marra et al. 2013). Evidence generated in LMICs on the usefulness of this strategy is urgently needed, because of the fundamentally different setting for the treatment of severe infections in resource-limited settings as outlined above. It is important to assess the efficacy and safety of the approach in hospitals with limited microbiological laboratory support in settings with differences in types of infections, patient case-mix, available resources, and standard of clinical practice (Lee, Kwa et al. 2020). In these settings, including in Bangladesh, concrete national guidelines to guide de-escalation or stopping of antibiotics in sepsis patients are often lacking or scarcely implemented (*Standard Treatment Guidelines (STG) on Antibiotic Use in Common Infectious Diseases of Bangladesh.* 2021). In addition to reducing antimicrobial drug pressure and reducing costs related to antibiotic usage, shortening intravenous antibiotic use could also potentially shorten ICU or hospital admission, freeing up scarce hospital and ICU bed capacity. A recent group of investigators proposed a systematic algorithm promoting the use of PCT thresholds to deescalate antibiotic treatment, but also emphasized the extreme lack of data coming from tropical LMICs settings (Lee, Kwa et al. 2020).

***Evolution of point-of-care PCT assays***

An important impediment of using sequential procalcitonin assessments for guiding antibiotic therapy in LMICs was the high cost of the assays, originally exceeding 30 USD, then reaching around 15-20 USD per measurement. Much cheaper quantitative point-of-care tests have recently come to the market. These tests come with single use cartridges of around 5 USD each and begin to have clinically acceptable accuracy data and margins of error. In addition, the costs will include the one-off purchase of the PCT-cartridge analyser, which are now available for around 5,000-10,000 USD. Although these costs have now been reduced considerably, it is important to assess how the costs of the assay compare with the potential reduction in costs of antibiotic usage if the procalcitonin guided de-escalation of antibiotics would be deployed. Showing cost-effectiveness of the strategy will be very important for its wider deployment strategy, of course provided it is safe and effective.

**Proposed study**

The proposed study is a randomised controlled, open-labelled, parallel, controlled trial of procalcitonin guided de-escalation of empirical antibiotic therapy in Bangladeshi patients hospitalized with suspected sepsis to evaluate the efficacy, safety, and cost-effectiveness of this strategy reducing the duration of antibiotic therapy.

# 6. OBJECTIVES AND OUTCOME MEASURES

| **Objectives** | **Endpoints** | **Timepoint(s) of evaluation of this outcome measure (if applicable)** |
| --- | --- | --- |
| **Primary Objective** | | |
| To assess the **efficacy** of daily procalcitonin measurements to reduce the length of antibiotic treatment in hospitalized patients with sepsis in a low-resourced setting | **Length of antibiotic treatment (LOT) during period:** number of days of antibiotic treatment during the study period . | Length of treatment will be measured from the day of enrolment to completion of the study follow up (28 days after hospital discharge). The primary outcome data for participants with recurrent infections will be censored at the time of recurrent infection diagnosis. |
| **Secondary Objectives** | | |
| 1. To assess if procalcitonin guided de-escalation reduces antibiotic **consumption** and days of therapy with antibiotics | Consumption of antibiotics expressed as the Defined Daily Dosage (DDD)  **Definition of DDD:** The basic definition for a defined daily dose is the assumed average maintenance dose per day for a drug used in its main indication in adults. Antibiotic use using the WHO DDD definition (<https://www.whocc.no/atc_ddd_index/>) will be determined for the most frequently prescribed Drugs in Bangladesh | DDD will be compared between study arms during the study period (day of enrolment to completion of the study follow up). |
|  | Days of therapy with antibiotics (DOT)  Definition of DOT: One DOT represents the administration of a single antibiotic on a given day regardless of the number of doses administered or dosage strength. If a patient receives more than one antibiotic it will be added on with DOT of the first antibiotic. (Dalton et al. 2015) | DOT will be measured from the day of enrolment to the day of stopping antibiotic(s). |
|  | Number of days of parenteral antibiotic during hospitalisation period | will be measured from the day of enrolment to the day of stopping of parenteral antibiotic during hospitalisation period |
|  | Length of antibiotic treatment (LOT) during hospitalisation period: number of days of antibiotic treatment during the hospitalisation period. | Length of treatment will be measured from the days of enrolment to stopping of antibiotic during hospitalisation period. |
| 2. To assess the safety of procalcitonin guided de-escalation of treatment | Overall mortality, mortality associated with recurrent infection.  Non-lethal recurrent infections.  Recurrent infections include culture positive relapse (failure of initial eradicative treatment for the original infecting strain), culture negative relapse (recurrence of initial symptoms after stopping of antibiotics and cultures negative), reinfection (infection with a different strain of the same species) and syndromic recurrence (recurrence of initial symptoms after stopping antibiotics with initial cultures negative and subsequent cultures either positive or negative or not done) . Recurrent infections are defined as growth of one or more of the initial causative bacterial strains (i.e., same genus, species and antimicrobial susceptibility) from a second sample taken from the same infection site after stopping of antibiotic therapy, combined with clinical signs and symptoms of infection. Superinfection is defined as the isolation from the same or another site of one or more pathogens different from that identified during the first infectious episode, together with clinical signs or symptoms of infection. | Overall mortality, defined as mortality during hospital stay and by day 28 after discharge.  Mortality associated with recurrent infection, defined as recurrence of initial symptoms (independent of culture results).  Non-lethal recurrent infection, defined as recurrence of initial symptoms (independent of culture results) during the study period (up to 28 days after hospital discharge). |
| 3. To assess the effect of procalcitonin de-escalation therapy on length of hospital stay | Duration of hospital stay (ICU/ general ward) | Duration of hospital stay (from day of hospital admission to the day of discharge) |
| 4. To determine the spectrum of antimicrobial resistance among bacteria identified in clinical specimens from Bangladeshi patients hospitalized with sepsis | Bacterial isolates from blood cultures will be identified, tested for antibiotic susceptibility. In a subset of antibiotic resistant isolates, genetic correlates of resistance will be assessed. | Blood cultures will be obtained at the baseline and when there is any readmission during suspected recurrent infection |
| 5. To compare the costs of the procalcitonin-guided de-escalation approach with the potential cost-savings in antibiotic usage | Direct medical costs of prescribed antibiotics, the PCT assay and hospital stay cost. | During hospitalization |

# 7. TRIAL DESIGN

**Trial design**

Randomised controlled, non-blinded, two-arm, parallel, phase 3 clinical trial comparing Standard practice to procalcitonin guided de-escalation of antibiotic therapy.

**Trial settings**

Trial site is Chattogram Medical College Hospital (CMCH), Bangladesh. CMCH is a tertiary care hospital with undergraduate and postgraduate teaching facilities. This tertiary hospital receives referrals from urban and rural areas of southern Bangladesh and has basic facilities for intensive care and haemodialysis.

**Duration of participant involvement**

Patients (16 to 65 years of age) admitted with suspected bacterial sepsis will be screened for eligibility. If the patient is eligible for inclusion, the research physician will propose the patient for involvement in the study. After obtaining informed written consent patient will be randomised to either the intervention or control arm of the study. After enrolment patient will be followed up daily until the day of discharge or death. After 28 days of discharge patient will be contacted for a follow-up assessment. The planned enrolment trial period is two years.

# 8. PARTICIPANT IDENTIFICATION

## 8.1. Trial Participants

The trial will recruit (16 to 65 years of age) male and non-pregnant female patients, admitted to the general medicine wards or ICU with a diagnosis of confirmed or suspected bacterial sepsis with an intention to start therapy with parenteral antibiotic(s). All study subjects must meet the applicable inclusion and exclusion criteria. All enrolled patients will have blood cultures collected and receive a parenteral antibiotic treatment. In the intervention group, de-escalation of antibiotic treatment will be guided by a daily quantitative PCT rapid test. In the control group, de-escalation will be according to the local guidelines.

**Operational definition of sepsis**

Sepsis should be defined as life-threatening organ dysfunction caused by a dysregulated host response to infection. For clinical operationalization, organ dysfunction can be represented by an increase in the Sequential [Sepsis-related] Organ Failure Assessment (SOFA) score of 2 points or more, which is associated with an in-hospital mortality greater than 10%. Septic shock should be defined as a subset of sepsis in which particularly profound circulatory, cellular, and metabolic abnormalities are associated with a greater risk of mortality than with sepsis alone. Patients with septic shock can be clinically identified by a vasopressor requirement to maintain a mean arterial pressure of 65 mm Hg or greater and serum lactate level greater than 2 mmol/L (>18 mg/dL) in the absence of hypovolemia.

**Working definition of sepsis**

In the background of suspected infection if the NEWS score corresponds to ≥ 5 or NEWS of 3 in any individual parameter will be considered as sepsis.

## 8.2 Inclusion criteria

- Participant, legally authorised person/ legal guardian willing and able to give informed consent for participation in the trial.
- Male or Female, aged 16 to 65 years
- Suspected or proven bacterial infection
- A positive sepsis screening National Early Warning Score (NEWS) equal or greater than 5 (**Table 1** or at least one criterion with a score of 3.
- Intention to start parenteral antibiotic therapy
- Within 24 hours of hospital admission

See table below for NEWS score and: <https://www.mdcalc.com/calc/1873/national-early-warning-score-news>

**Table 1.** NEWS Scoring table for screening

* National Early Warning Score. Scoring system that includes seven physiologic variables or vital signs, i.e. respiration rate, oxygen saturation, any supplemental oxygen, temperature, systolic blood pressure, heart rate and level of consciousness (as measures by the AVPU – Alert, Voice, Pain, Unresponsive). These parameters are routinely collected as part of patient care. The higher the NEWS, the more likely that the patient is developing a critical illness. A score of five or higher has been recommended as an indicator to screen for sepsis (NEWS 2020, Pairattanakorn, Angkasekwinai et al. 2021).

8.3 **Exclusion Criteria**

The participant may not enter the trial if ANY of the following apply:

- Patients with suspected/documented tuberculosis, parasitic infection (including malaria or visceral leishmaniasis), or viral infections (i.e., COVID19, dengue, HIV)
- Pregnancy
- Intended for a short stay in ICU or general ward (such as post-operative)
- Patients requiring a predefined long course of antibiotic therapy (such as endocarditis, osteomyelitis, lung abscess, liver abscess, septic arthritis)
- Immunocompromised patients, including as severe neutropenia (< 500 cells/ml), transplant recipients, on prolonged corticosteroid treatment, chemotherapy or disease modifying immunomodulatory medications
- More than 48 hours of parenteral antibiotic use
- Surgical patients, including patients with a surgical septic source or patients requiring source control, i.e. abscess drainage
- Moribund patients or patients receiving end of life care
- Previous enrolment in PROCALBAN
- Conditions accompanied with a systemic inflammatory state, including pancreatitis, cardiogenic shock, severe trauma

**8.4 Consort Diagram**

The trial will be reported according to the CONSORT 2010 statement (Schulz, Altman et al. 2010).

Proposed flow diagram, including the expected numbers of patients (based on assumptions)

# 9 TRIAL PROCEDURES

**Engagement with treating hospital physicians**

Before start of the study and quarterly during the study, there will be several sessions organised with the treating physicians at CMCH to explain the study and their involvement. This will require providing the physicians with detailed scientific background on the use of sequential PCT assessment for de-escalation of antibiotic treatment. The sessions will emphasize the importance of compliance with the antibiotic treatment advice based on the PCT results in the intervention arm. In addition, it will be clearly communicated that the treating physician has the ultimate decision to continue or discontinue antibiotic treatment, which can be based on additional clinical or laboratory parameters. The sessions will include an opportunity to raise concerns about following the advice to stop antibiotic treatment based on PCT concentrations, and to discuss a variety of scenarios in which reaching the cut-off value for PCT concentration will or will not be an appropriate guide to discontinue antibiotic therapy. The format of the sessions will be power-point presentations by the study team, question and answer sessions, and scenario-based group discussions. An information booklet including study protocol de-escalation therapy and literature on PCT-based de-escalation of therapy will be prepared for all participating physicians at CMCH. In addition, engagement meetings will be organized with ward senior medical staff, departmental heads and hospital management in order to provide the necessary background of the study, and emphasize the importance of collaboration on the study.

**Screening**

Initial screening of study participants will be done by the physician on duty on patients aged between 16-65 years presenting with suspected or confirmed features of infection. Intention to start parenteral antibiotics will be assessed based on prescriptions by the treating team. The National Early Warning Score (NEWS) will be used to identify suspected sepsis. If the aggregate NEWS score is ≥ 5 or 3 in any individual parameter of NEWS score eligibility for inclusion in the study will then be assessed by the study physician. If patient fulfils all other inclusion and exclusion criteria the study physician will perform the consenting procedure. After informed written consent is obtained from the patient, the patient will be randomised to either the intervention or control arm. A positive blood culture for a bacterial pathogen will confirm a diagnosis of bacterial sepsis, but this is not a prerequisite for enrolment in the trial.

Schedule of sampling and procedures is detailed in **Appendix B.**

## 9.1 Recruitment

Patients between the ages of 16 and 65 who visit the emergency room, outpatient general medicine clinic, or are admitted to the general medicine wards or intensive care unit at CMCH will undergo initial screening by the attending physician. If the physician intends to initiate intravenous antibiotics or suspects a severe infection based on clinical judgment, and the patient's NEWS score meets specific thresholds for urgent assessment (NEWS ≥ 5) or urgent ward-based assessment (e.g., NEWS of 3 in any individual parameter, according to the NEWS Score 2020), they will inform the study physicians. The study physicians will then evaluate the patient's eligibility based on the appropriate criteria.

Once eligibility is confirmed, the study will be explained to the patient, their relative, or legal guardian. The research physician will then conduct the consent procedure, which involves providing information about the study through the participant information sheet and obtaining written informed consent. Any reasons for exclusion, such as refusal to provide informed consent, will be recorded in the 'Screening and Enrolment Log'. After obtaining consent, the patient will be enrolled in the study and randomly assigned to either the experimental arm (procalcitonin) or the control arm (standard therapy). Initially, data collection will be conducted using paper CRFs until the online database is prepared for the study. Subsequently, electronic tablets will be utilized to collect data once the online database is fully operational and functional.

(see section 9.3). Overall recruitment and screening flow chart are described in **Appendix A**.

**Table 2.** NEWS threshold and triggers

| Aggregate score 0-4 | Low | Ward-based response |
| --- | --- | --- |
| Red score  Score of 3 in any individual parameter | Low- medium | Urgent ward-based response |
| Aggregate score 5-6 | Medium | Key threshold for urgent response |
| Aggregate score 7 OR more | High | Urgent or emergency response |
| Source: National Early Warning Score systems that alert to deteriorating adult patients in hospital  Medtech innovation briefing [MIB205] Published: 18 February 2020 | | |

## 9.2 Informed Consent

The participant or legally authorised person must personally sign and date the latest approved version of the Informed Consent Form (ICF) before any trial specific procedures are performed. It will be clearly stated that refusal to participate will not jeopardise subsequent treatment of infection.

A written version of the Participant Information Sheet (PIS) and ICF will be presented and verbally explained to the patients, legal guardian/ legally authorised person detailing no less than: the exact nature of the trial; what it will involve for the participant; the implications and constraints of the protocol; the known side effects and any risks involved in taking part. The study description will be explained in Bangla, or other appropriate local dialect as needed, and a translated ‘Participant Information Sheet’ paper copy will be provided to the patients, legal guardian/ legally authorised person. It will be clearly stated that the participant is free to withdraw from the trial at any time for any reason without prejudice to future care, without affecting their legal rights and with no obligation to give the reason for withdrawal.

The Investigator will ensure that the consent procedure is comprehensive yet straight forward, and will not delay start of antibiotic treatment. However, due to time-sensitivity of study assessments, the participant, legal guardian/ legally authorised person will be allowed a maximum of 1 hour to consider the information, and the opportunity to question the Investigator, their local physician, or other independent parties to decide whether they will participate in the trial. The enrolment procedure for the study will not delay the start of antibiotic therapy prescribed by the attending physician. Written Informed Consent will then be obtained by means of participants (or witness if illiterate) dated signature (or thumb print plus dated signature of literate witness if legal guardian/ legally authorised person is unable to write) and a dated signature of the person who presented and obtained the Informed Consent. The person who obtained the consent must be suitably qualified and experienced and have been authorised to do so by the Principal Investigator. A copy of the signed Informed Consent will be given to the participant. The original signed form will be retained at the trial site in the ‘**Enrolment Log’** binder to de-identify the ‘Case Report Form’ (CRF).

For severely ill, the legal guardian/ legally authorised person of patients who are severely ill at the time of recruitment would be asked for written agreement for study participation. Before any study-specific procedures are carried out, the legal guardian/ legally authorised person must physically sign and date the most recent approved version of the Informed Consent Form (ICF). An impartial witness will be asked to countersign to prove their consent if they are an illiterate legal guardian/ legally authorised person.

## 9.3 Screening procedures

Initial screen will be performed as outlined in Section 9.0. Patients identified through initial screening will be assessed for eligibility using the inclusion and excluding criteria summarized in section 8.2. Each febrile patient will have a malaria rapid diagnostic test performed. Patients with malaria will be excluded. Other diagnostic tests for infections listed in the exclusion criteria, e.g. dengue virus infection, will be performed as clinically indicated. The maximum duration of time allowed between screening and randomization will be 1 hour.

A ‘Screening CRF Form’ will be completed for all subjects with suspected infection and intention to start parenteral antibiotics by the treating team. A NEWS ≥ 5 or at least one criterion with a score of 3 will determine further eligibility.

Two separate databases will be maintained for the screening and enrolment processes. The screening database will include information on all patients who undergo screening, including those who do not meet the eligibility criteria. Any instances of screening failure will be recorded in this database, along with the reasons for exclusion.

On the other hand, the enrolment database will only include data of eligible participants who have provided written informed consent. This database will exclusively contain information about participants who have agreed to participate in the study and meet the necessary criteria for enrolment. If patients do not meet inclusion or meet exclusion criteria, they will be immediately referred to the ward doctor along with any results of initial screening assessments.

**9.3.1 Demographic and medical history**

Abbreviated demographic, symptom and medical history related to inclusion and exclusion criteria will be obtained, i.e. age, focal symptoms of infection, recent antibiotic therapy.

**9.3.2. Physical exam and vital signs**

Abbreviated physical examination related to the NEWS parameters will be conducted by a research physician, i.e. blood pressure, respiratory rate, oxygen saturation, temperature, heart rate, and AVPU score.

## 9.4 Randomisation and blinding

We will seek to enrol all patients with suspected sepsis who give consent as soon as the patient is diagnosed. Enrolment will coincide with or occur shortly before the start of parenteral (usually intravenous) antimicrobial therapy and all other applicable urgent treatments, to ensure no treatment delays. After eligibility is confirmed, the patient will be randomised to either the experimental (procalcitonin) arm or control (standard therapy) arm. Randomisation and treatment allocation will be determined by a central computerized binary sequence number generator in a 1:1 ratio with pre-determined block sizes by the study statistician. The study statistician will design the randomisation schedule. The subject randomisation number will be on the envelope as well as in the insert inside the envelope. The trial staff will be opening the envelopes sequentially to eligible trial participants and allocate the arm that is found in the insert of that envelope. The allocated arm will be added on the eCRF and other applicable documents. Investigators and patients will not be blinded to intervention randomisation.

## 9.5 Clinical procedures

### 9.5.1 Clinical assessment during baseline and initial procedures

***Demographic and medical history***

Basic demographic and epidemiological data (i.e., age, sex, village, Upazila, district, socio-economic condition) and a full medical history such as clinical presentation, comorbid conditions such as diabetes, hypertension, chronic kidney disease, chronic liver diseases, ischemic heart disease, heart failure, malignancy, or any other chronic medical condition will be obtained and recorded in the CRF by the research assistant. All prescribed or over-the-counter drugs, and traditional medications used within the last 7 days and any drug allergies will also be recorded in the CRF.

***Physical exam and vital signs***

Upon enrolment, a physical examination will be conducted by a research physician, Glasgow coma scale (GCS), pulse, blood pressure, respiratory rate, oxygen saturation, perfusion index aural temperature, weight, height, capillary refill time, capillary blood glucose, oxygen requirement and remainder of the physical exam will be recorded in the CRF.

***Antimicrobial cultures and start of antibiotics***

All patients enrolled in the study will have a blood culture collected (detailed below) and/or additional microbiological testing as indicated by the treating physician. Patients will be started on parenteral antibiotic treatment as soon as microbiological cultures are taken. In the event antibiotic treatment was already started (but less than 48 hours) before a blood culture was taken, a blood culture will still be collected, but it will be recorded that this was done under antibiotic therapy. The choice of antibiotic treatment will be at the discretion of the treating physician as per [[local guidelines](https://amr.cdc.gov.bd/wp-content/uploads/2018/10/STG-guideline-for-antimicrobial-use-version-1.0-date-1-december21.pdf)](https://amr.cdc.gov.bd/wp-content/uploads/2018/10/STG-guideline-for-antimicrobial-use-version-1.0-date-1-december21.pdf) . All antibiotic administered will be recorded in the CRF.

***Blood sampling***

Blood sampling after enrolment is detailed in Section 9.8.

### 9.5.2 Clinical assessments during hospitalization

Enrolled patients will be assessed daily during hospitalization until day of discharge as follows:

- Daily symptom questionnaire for complications or clinical deterioration/recurrent infection
- GCS, blood pressure, heart rate, capillary refill time, respiratory rate, and oxygen saturation, perfusion index, , oxygen requirement, temperature (aural) measurement daily up to discharge from the hospital. A capillary blood glucose will be measured at baseline and if clinically indicated during daily assessment.
- Daily physical examination
- Daily recording of antibiotic administration (name, route, dosage, and duration) and concomitant medications in CRF

### Recording of adverse events (AEs), and serious adverse events (SAEs)- specifically, the study physicians will aim to detect any complications, clinical deterioration and side effects related to antibiotic use, i.e. diarrhoea, allergic reaction and severe skin reaction, deaths.

### 9.5.3. Imaging

Imaging will be done if clinically indicated. A systematic 12-zone lung ultrasound will be performed on all study patients at baseline (D0H0) following international guidelines.

### 9.5.4. Follow up visit

**Day 28 after discharge**: (either in person or over the telephone)

During physical follow up visit following assessments will be done:

- Outcome (alive or dead; any history of re-admission due to recurrent infection)
- Standardized symptom questionnaire
- Vital signs and physical examination will be performed if recurrent symptoms are present at in person follow up.
- Current and/or interval use of antibiotics

## 9.6. Control group and intervention group procedures

In all patients, antibiotics will be started based on a clinical suspicion of infection or microbiological evidence of infection. The decision regarding timing of antibiotic initiation and of type of antibiotic prescribed will be at the discretion of the treating physician and in accordance with CMCH and/or [National guidelines](https://amr.cdc.gov.bd/wp-content/uploads/2018/10/STG-guideline-for-antimicrobial-use-version-1.0-date-1-december21.pdf) .

**Intervention arm (procalcitonin test group)**

If a patient is randomised for the procalcitonin group, procalcitonin will be measured in the D0H0-serum sample (D0H0: as close to start antibiotics as possible, at least within 2 hrs). The treating physician will receive daily procalcitonin values until the patient is discharged from the ICU or hospital, or until two days after all systemic antibiotics have been stopped or two days after providing nonbinding advice to stop antibiotics whichever occurs earlier. Along with daily procalcitonin values a non-binding advice will be generated to consider stopping the prescribed antibiotics based on procalcitonin results. An illustration of trial intervention based on PCT results given in **Figure 1.** The treating physicians will have an option to continue, stop or restart antibiotic therapy at any time during hospitalisation, based on their own judgement and independent of reaching the target PCT values. However, if the stopping advice is not followed, the treating physician rationale will be recorded in the CRF.

For specific infections, such as culture proven typhoid fever or melioidosis, a minimum duration of antibiotic treatment is recommended in the local guidelines (e.g. Standard Treatment Guidelines (STG) on Antibiotic Use in Common Infectious Diseases of Bangladesh), which will be adhered to in this study. For this purpose, [an online application (STG-DGHS) developed by the Directorate General of Health Services (DGHS) containing standard treatment guidelines of common infections](https://play.google.com/store/apps/details?id=stgapp.cdc.gov.bd) of / local antibiotic guidance will be provided to the treating physicians.

**Control arm (no procalcitonin test group)**

If a patient is randomised to the control group, no procalcitonin measurements will be performed. The patient will be managed according to the treating physician’s discretion. The decision about antibiotic discontinuation will be based on clinical expertise, national guidelines, and any microbiology results that might be available. If a treating physician recommends a non-study related PCT test to be performed (in hospital or external laboratory), this result will be recorded in the CRF.

**Patients randomised to intervention group, but initial PCT level is below 0.5ng/ml.**

If the initial PCT level is <0.5ng/ml we will assume systemic bacterial infection is unlikely. In these patients we will perform three consecutive daily PCT assays including the first one (i.e. D0H0, D1H24, D2H48) and if the three consecutive tests are found < 0.5 ng/ml , the treating physician will receive non-binding advice to discontinue antibiotic treatment

**Figure 1.** Trial intervention

**Table 3.** Likelihood of bacterial infection based on PCT levels.

| Level of PCT (ng/ml) | Likelihood of bacterial infections |
| --- | --- |
| < 0.05 | Healthy adult |
| 0.05 to <0.5 | Systemic infection is unlikely although localised infection is possible |
| 0.5 to <2 | Systemic infection is possible but other conditions (e.g., major trauma, recent surgery, severe cardiogenic shock) may also induce PCT rises. |
| 2 to <10 | Systemic infection is likely |
| ≥ 10 | High likelihood of severe bacterial sepsis or septic shock |
| This interpretation assumes a clinically compatible syndrome and the absence of other cause of procalcitonin elevation  Source: (Samsudin, Vasikaran 2017). | |

## 9.7 Assessment of Endpoints

***Length of antibiotic treatment***

Length of antibiotic treatment will be defined as number of days of antibiotic treatment during the study period (till day 28 after hospital discharge).

***Consumption of antibiotics expressed as the Defined Daily Dosage (DDD)***

Defined daily dosage (DDD) is defined as the basic definition for a defined daily dose is the assumed average maintenance dose per day for a drug used in its main indication in adults. Antibiotic use using the WHO DDD definition (<https://www.whocc.no/atc_ddd_index/>) will be determined for the most frequently prescribed Drugs in Bangladesh.

***Days of therapy with antibiotics (DOT)***

Days of therapy with antibiotics (DOT) is defined as follows: the DOT represents the administration of a single antibiotic on a given day regardless of the number of doses administered or dosage strength. If a patient receives more than one antibiotic it will be added on with DOT of the first antibiotic (Dalton et.al., 2015). For the purposes of this study this will include both parenteral and oral antibiotics.

***Recurrent infections***

Recurrent infections will be suspected if patient develops any clinical features of infection after stopping of antibiotics and within the study period (until 28 days after hospital discharge). Patients with recurrent infections and superinfections (table 4) will not be included and randomized for a second time. Recurrent infections will include: (i) culture positive relapse (failure of initial eradicative treatment for the original infecting strain), (ii) culture negative relapse (recurrence of initial symptoms after stopping of antibiotics and cultures negative), (iii)reinfection (infection with a different strain of the same species) and (iv) syndromic recurrence (recurrence of initial symptoms after stopping antibiotics with initial cultures negative and subsequent cultures either positive or negative or not done). Recurrent culture positive infections are defined as growth of one or more of the initial causative bacterial strains (i.e., same genus, species and antimicrobial susceptibility) from a second sample taken from the same infection site after stopping of antibiotic therapy, combined with clinical signs and symptoms of infection. Superinfection is defined as the isolation from the same or another site of one or more pathogens different from that identified during the first infectious episode, together with clinical signs or symptoms of infection.

**Table 4**: Recurrent infection and superinfection

|  | Baseline | Subsequent events | Strain of isolates | Comments |
| --- | --- | --- | --- | --- |
| Culture positive relapse | Culture positive | Culture positive | Same strain and same species |  |
| Culture negative relapse | Culture positive | Culture negative | Not applicable | Recurrence of initial symptoms after stopping of antibiotics |
| Reinfection | Culture positive | Culture positive | Different strain of the same species |  |
| Syndromic clinical relapse | Culture negative | Culture negative or positive or not done | Not applicable | Only reappearance of initial symptoms |
| Superinfection | Culture positive | Culture positive | Same or another site of one or more pathogens different from that identified during the first infectious episode |  |

***Safety endpoints***

Mortality will be assessed as overall mortality, mortality associated with recurrent infection and non-lethal recurrent infections, as defined above. For overall mortality, any patient who is discharged home to die will be recorded as a fatal case.

***Duration of hospital stay***

Duration of hospital stay will be measured in days calculated from hospital admission hour until the hospital discharge (rounded by 24-hour quarters, i.e. 00:00-06:00 = 0.25 days; 00:00-12:00 = 0.5 days; 00:00 – 18:00 = 0.75 days; 00:00 – 24:00 = 1 day).

***Economic cost analysis***

The economic analysis will focus on a comparison of direct medical costs of antibiotic therapy and hospital stay between the control and intervention groups. To calculate per patient cost of antibiotic therapy for the intervention group, the total cost of antibiotic treatment will be added to cost of procalcitonin test, and the resulting figure will be divided by the number of patients in the intervention group to determine the per patient treatment cost. In the control group, the total cost of antibiotic therapy will be divided by the number of patients in the control group to determine the per patient treatment cost. The sample size number of patients randomly assigned to each of the two groups is 266. Standard maximum retail prices of the antibiotic fixed by the producers and an average PCT testing charge estimated from the invoice will be used. The inpatient costs will be calculated based on hospital length of stay multiplied by the average inpatient cost per day, which will be obtained from pre-study CMCH data.

The indirect medical expenditures will also be assessed by the estimated total reduction in income loss for the patients and caregivers. Inpatient days will be multiplied by self-reported average wage/income per day as an estimate of income loss. Information on incomes/wages and other variables will be directly collected from the patient/caregiver using a short, structured questionnaire at some point during the intervention period.

## 9.8 Sample Handling

### 9.8.1 Sample handling for trial purposes

All samples will be processed as per study standard operating procedures at CMCH and Apollo Imperial Hospital. Samples will be stored at appropriate temperatures, which will be monitored daily. Samples requiring shipping will be stored in -80 degree Celsius freezer / Liquid Nitrogen until shipping.

### 9.8.1.1 Blood sampling on enrolment/baseline (H0)

At enrolment, venous blood will be collected for all participants for the following:

**Table 5**. Sample handling

| **Sl** | **Specimen** | **Tube/container** | **Amount/volume** | **Assay** |
| --- | --- | --- | --- | --- |
| 01 | Blood | Tube:  K2-EDTA tube (2mL) | Blood volume= 2 mL  Whole blood for complete blood count (CBC) | Haematology Analyser (Sysmex XN-2000) at Chevron Laboratory |
| 02 | Blood | Tube:  Sodium citrate 0.4 ml (2 mL tube) | Blood volume = 1.6 mL | Erythrocyte sedimentation rate (ESR) machine (Diesse Ves-Matic Cube 80) at Chevron Laboratory |
| 03 | Blood | Tube:  Clot activator tube (4 mL) | Blood volume = 2.5 mL  (a) 0.5 mL serum for creatinine and total bilirubin  (b) 0.2 mL serum will be used for procalcitonin test within 2 hours of enrolment (experimental group only)  (c) 0.5 mL serum for storage  For follow up PCT testing and serum storage 1 mL of blood is collected daily for approximately 3-7 days | Biochemistry Analyser  (Beckman Coulter AU 680) at Chevron Laboratory, parameters will be used to calculate SOFA score.  Serum PCT (experimental group) by IMMU F6 CLIA. Analyser (MedCaptain, China) |
| 04 | Blood | Bottle:  Blood culture bottle (10 mL X 2) | Blood volume = 16-20 mL  Whole blood will be collected from two separate sites. | BacTec Fx40 at Apollo Imperial Hospital, Chattogram |

During screening, a malaria rapid diagnostic test (RDT) will be done. Approximately 80 microlitre (0.08mL) of blood will be collected through finger prick from all patients with fever.

Other cultures, i.e. urine, abscess, sputum, CSF, stool, wound swabs, will be performed if clinically indicated as per treating physician and testing performed at Apollo Imperial Hospital Microbiology laboratory.

**9.8.1.2. Blood sampling during hospitalization**

***Procalcitonin assay***

Daily serum procalcitonin assay will be assessed daily until two days after de-escalation of antibiotics in the experimental arm only. No placebo or alternate assay will be performed in the control group beyond baseline. Whole blood sample (as per Section 10) collected PCT and quantified via Chemiluminescence Immune Assay (CLIA) method using an Immuo F6 analyser (MedCaptain Company, China). The research laboratory staff will perform the assays at the CMCH Research Laboratory.

**Blood sampling during follow up**

Follow-up laboratory assessments including haematology, biochemistry or microbiological tests, will be performed at the discretion of the treating physician.

**Blood tests for microbiological testing**

Blood cultures and antibiotic susceptibility testing will be performed at Apollo Imperial Hospital Microbiology Laboratory, Chattogram, Bangladesh.

### 9.8.2 Sample handling for control arm

The control arm will receive at the same baseline laboratory and microbiological testing as the intervention group, apart from the Procalcitonin assay.

## 9.9 Blood volumes and analysis summary

The blood volumes for the protocol mandated tests are details in the study schedule (Table 5**)**. Maximum blood volumes are presented below for 28 days of follow up. The maximum blood volume is the total amount taken for the duration of the study.

The maximum blood volume cumulative over 28 days after discharge will be approximately 27- 31 mL, which is less than 10% of total blood volume generally recommended as a maximum volume to be taken over 8 weeks.

The locations and methodologies of all investigations have been chosen as all the laboratories have quality-controlled procedures, use validated assays and deploy fully trained personnel.

## 9.10 Early Discontinuation/Withdrawal of Participants

During the trial a participant may choose to withdraw early from the trial treatment at any time. This may happen for several reasons, including but not limited to:

- Inability to comply with trial procedures
- Participant decision

Participants may choose to stop study assessments, without consequences for their medical treatment. The patient may also to withdraw from the study. In such case the patient will be asked for permission to use the study data collected until the time of withdrawal.

In addition, the Investigator may discontinue a participant from the trial treatment at any time if the Investigator considers it necessary for any reason including, but not limited to:

- Ineligibility (either arising during the trial or retrospectively having been overlooked at screening)
- Significant protocol deviation
- Significant non-compliance with the trial requirements
- An adverse event which requires discontinuation of the trial or results in inability to continue to comply with trial procedures

The type of withdrawal and reason for withdrawal will be recorded in the CRF.

If the participant is withdrawn due to an adverse event, the Investigator will arrange for prompt clinical management by the attending physician.

## 9.11 Definition of End of Trial

The end of trial is the point at which all the data including data of follow up visits has been entered and queries resolved.

# 10 TRIAL INTERVENTIONS

The intervention is a daily assessment of serum procalcitonin level to actively guide de-escalation of antibiotics. In the intervention group serum procalcitonin level will be measured at baseline and then daily until discharge or two days of stopping antibiotic therapy or until two days of providing non-binding advice to stop antibiotic. First PCT measurement will be done at recruited in the study; within 2 hours of enrolment. At 72 hours, the treating physicians will be advised (as per protocol) to stop the prescribed antibiotics if the PCT level decreases by ≥80% from peak concentration and/or decreases below an absolute value of 0.5 ng/ml or three consecutive PCT results including the baseline remain <0.5 (table 6). The treating physicians will be provided a copy of the PCT results for the hospital chart along with print-out advice regarding continuation of antibiotic therapy. The treating physicians will hold the ultimate decision regarding continuation or discontinuation of antibiotics. If the treating physicians decide to continue antibiotics, the reason for doing so will be recorded in the CRF.

The control group will not receive any advice regarding antibiotic de-escalation/discontinuation. Duration of antibiotics in the control group will be determined by the treating physicians as per CMCH and/or [National Guidelines](https://amr.cdc.gov.bd/wp-content/uploads/2018/10/STG-guideline-for-antimicrobial-use-version-1.0-date-1-december21.pdf)

**Table 6**. PCT levels for non-binding advice to treating physicians

| Baseline PCT level ng/ml | Decision on stopping antibiotic treatment | Minimum time required to provide non-binding advice |
| --- | --- | --- |
| >2 | PCT levels decreased to 80 % of its peak value or has reached a value of below an absolute value of 0.5 ng/ml, whichever comes first | 72 hours from the baseline test |
| 0.5- 2 | below 0.5 ng/ml | 72 hours from the baseline test |
| < 0.5 | <0.5 we will advise systemic bacterial infection in unlikely. In these patients we will perform three consecutive daily PCT assays at D0H0, D1H24, D2H48 andif the three consecutive test results are <0.5 ng/ml , the treating physician will receive non-binding advice to discontinue antibiotic at 48 hours. The antibiotic should be stopped if the patient’s vitals are stable and patient isafebrile. | 48 hours from the baseline test |

### 10.1 Investigational lab testing

***Procalcitonin assay***

Daily serum procalcitonin assay will be done in the experimental arm only. Whole blood sample (1 ml) collected by using a 4mL serum collection tube. PCT will be quantified via Chemiluminescence Immune Assay (CLIA) method using an Immuo F6 analyser (MedCaptain Company, China). Research laboratory staff will perform the assays at the CMCH Research Laboratory such that results will be available in real time. The Medcaptain Immuo F6 analyser PCT Chemiluminescence Immune Assay (CLIA) has CE certification for clinical use. Validation of the Immuo F6 analyser PCT Chemiluminescence Immune Assay (CLIA) will be performed by MORU clinical laboratory technician at CMCH prior to the start of the study then every 6 months for the duration of the study and results will be compared to Chevron Ctg. Ltd ISO accredited laboratory.

***Microbiology assays***

**Blood culture**s:

Blood cultures will be incubated in automated BacTec Fx40 system at Apollo Imperial Hospital, Chattogram, Bangladesh. Venous whole blood (2 times 8-10 ml) will be collected aseptically into two culture bottles; one set of culture means two bottles from different peripheral venepuncture sites. Inoculated culture bottles will be barcoded and incubated in an automated culture system. When the system indicates growth, the positive blood culture bottles will be removed from the incubator for Gram staining and subculturing onto culture plates including blood agar, MacConkey agar, and Chocolate agar. Identification of isolated bacteria will be performed using conventional biochemical tests or automated identification (MicroScan WalkAway 96 Plus). Blood culture bottles will be incubated for 5 days before discarding and reporting as negative growth.

Urine, sputum, tracheal aspirate or pus cultures will only be performed if indicated at the discretion of the treating physician. However, culture results will be recorded in the CRF.

For completeness we provide the procedures here.

**Urine cultures:**

Approximately 10-15 ml of clean catch mid-stream urine in a sterile wide mouthed urine pot or in case catheterized patients from catheter tubes after clamping (not from urine bags) will be collected. 10µl of uncentrifuged urine will be inoculated onto Blood agar, MacConkey agar and HiChrome UTI agar. If there is delay in transporting the sample to Apollo Imperial Hospital, the sample will be kept at 2-4^0^C. Urine sample will be centrifuged at 3000 rpm for 10 minutes and wet mount prepared to examine for leukocytes cells (>10 leukocytes per high-powered field. is considered as significant pyuria). Inoculated agar plates will be incubated for 24 hours at 37°C aerobically, then assessed for growth and colony count (if any growth). Colony count will be considered significant if >10^3^ to 10^5^ for single or two types of colonies. If there is >3 types of colonies, this will be considered as contamination and repeat sample may be collected for culture if the patient is still clinically unwell despite initial therapy. All participants will undergo urine dipstick testing, and if leukocytes are detected, further examination through urine microscopy will be conducted. If the leukocyte count exceeds 10 per high-power field (HPF) in microscopy, urine cultures will be performed.

**Sputum culture:**

Approximated 5 ml of sputum (not saliva) will be collected for culture; the quality of the sample will be assessed by microscopy (wet preparation and gram-stained smear). Presence of polymorphonuclear cells (>10 per low-powered field) and squamous epithelial cells (<10 per low-powered field) will be considered as the minimum acceptable criteria for cultures to be set up on Blood agar, MacConkey agar, Chocolate agar. Plates will be incubated at 37°C aerobically and in a CO2 incubator for 48 hours.

**Tracheal aspirate culture:**

Specimens will be examined for the presence of polymorphonuclear cells (>10 per low-powered field) and any organisms under oil immersion microscopy. Specimens meeting these criteria will be acceptable for culture and inoculated in Blood agar, MacConkey agar, Chocolate agar and incubated for 24 hours at 37°C.

**Abscess/purulent discharge culture:**

Suspected abscess fluid will be aspirated with needle and syringe for culture if possible. Aspirated fluid will be collected aseptically into sterile culture cups. Aspirated fluid will be inoculated onto Blood agar, MacConkey agar, and Chocolate agar plates and incubated for 24 hours at 37°C.

**Antibiotic susceptibility testing:** All isolated pathogenic strains will undergo antibiotic susceptibility testing by Kirby-Bauer disk diffusion as per Clinical and Laboratory Standards Institute (CLSI M100 2023). The panel of antibiotics tested for susceptibility vary according to the isolate. All tests will be performed on Muller-Hinton agar media except fastidious organisms like *H. influenzae, N. meningitidis, S. pneumoniae, S. pyogenes, Enterococcus* for which enriched media will be used. Prior to inoculation, a bacterial suspension having visually equivalent turbidity to 0.5 McFarland standards will be prepared. A swab stick will be dipped into the standardized suspension then taken out and squeezed on the wall of the test tube to discard extra suspension. The surface of dried agar dish will then be uniformly inoculated by the cotton swab. Kirby-Bauer discs are then applied to the agar surface ensuring full contact of the disc with the agar. After disc placement, the agar plates will be rapidly incubated within 15 minutes (to avoid falsely enlarged zones of inhibition) at 35°C to 37°C for 24 hours. Plates will then be assessed by measuring the zone of inhibition (including the diameter of the disc). Inhibition zones will be measured in millimetre (mm) using a ruler over the surface of the plate with the lid open and graded as Sensitive (S), Intermediate (I) and Resistant (R) according to the reference zone of inhibition for the antibiotic (CLSI M100 2023).

***Genetic study***

Blood bacterial isolates showing resistance to 2 or more applicable antibiotic classes (multidrug resistant or MDR) will be stored at the Apollo Imperial Hospital for follow-up assessments. From a selection of MDR isolates, DNA will be extracted for molecular characterization. This will include the assessment of resistance genes, or in case reinfection in an individual patient or a local outbreak is suspected, whole genome sequencing. According to the type of assay, the appropriate laboratory will be identified. Serum samples from the collected samples will be stored in a -80 degree C freezer for future advanced molecular studies.

### 10.1.1. Blinding of PCT assay

No blinded.

### 10.1.2. Storage of procalcitonin kit and reagents

Storage of PCT Kits and reagents will be done according to the manufacturer's instructions.

### 10.1.3. Accountability of the Trial Intervention

All PCT tests will be numbered and usage logged by the laboratory staff.

## 10.2  Other Treatments (non-IMPS)

Not applicable.

## 10.3 Other Interventions

Not applicable.

# 11 SAFETY REPORTING

The procedures of this clinical studies are part of routine clinical care, except for the small blood sample used for assessment of PCT in the intervention group.

However, one of the main objectives of the study is to assess whether PCT-guided de-escalation of antibiotic therapy is safe, and thus not result in increased morbidity through recurrent infections, clinical deterioration after stopping antibiotic therapy, prolonged hospital stay, or increased mortality. A symptom questionnaire will be performed daily during hospitalisation and at each subsequent visit to the health care facility, to aid in the identification of adverse events. A data safety and monitoring board (DSMB) will have full access to the safety outcome data of the study, and the DSMB will receive the unblinded trial results after first 40 patients enrolled for safety analysis.

Clinical signs or deterioration that emerge following study initiation will not be categorized as adverse events (AEs). Nevertheless, any information pertaining to clinical deterioration, including the onset of pulmonary oedema, the requirement for invasive mechanical ventilation (MV), non-invasive ventilation (NIV), or high-flow nasal cannula therapy (HFNT), shock, initiation of inotrope-vasopressor, , spontaneous bleeding, renal failure, and initiation of renal replacement therapy (RRT), occurring within two days after discontinuing antibiotics and throughout the hospitalization period, will be documented and presented during the Data and Safety Monitoring Board (DSMB) meeting.

## 11.1 Assessment results outside of normal parameters as AEs and SAEs

In this study, no investigational medicinal product will be given to subjects. This protocol defines an AE as any untoward medical occurrence in a subject undergoing a study related procedure or intervention, which is not already defined as a secondary endpoint. Any medical condition or abnormal laboratory value that is present at the time that the patient is screened will be considered as baseline and not reported as an AE. For all patients, all adverse events will be recorded from enrolment until withdrawal or discharge from hospital. AEs thought related to the study protocol and serious adverse events (defined below) will be recorded. Recurrent infection will be reported in separate ‘Recurrent infection CRF’ as opposed to being considered as an AE since this is a secondary endpoint.

**Adverse events:**

1. Septic thrombophlebitis
2. Catheter-associate urinary tract infection
3. Antibiotic related severe side effects- diarrhoea, allergic reaction, skin reaction

**Serious adverse events:**

1. **ICU referral**

**2. Death**- All deaths, whether related to the study intervention or unrelated, such as complications arising from sepsis or treatment, will be documented and reported as serious adverse events (SAEs).

## 11.2 Procedures for Reporting Adverse Events

The following information will be reported on the CRF: description, date of onset and end date, severity, assessment of relatedness to trial procedure and action taken. Follow-up information should be provided as necessary.

The severity of events will be assessed on the following Common Terminology Criteria for Adverse Events (CTACE) grading table (version 5.0). :

Non-serious AEs considered related to the trial will be followed up either until resolution, or the event is considered stable as judged by a medically qualified investigator or the Sponsor.

All SAEs must be reported on the SAE Reporting Form to the Sponsor or delegate immediately or within 24 hours of Site Study Team becoming aware of the event being defined as serious. The DSMB has the authority to decide on the selective reporting of serious adverse events (SAEs) if deemed necessary.

### 11.2.1. Procedure for immediate reporting of Serious Adverse Events

- Site study team will complete an SAE report form for all reportable SAEs.

Where the SAE requires immediate reporting, the SAE report form will be scanned and emailed to PI and site PI immediately i.e., within 24 hours of site study team becoming aware of the event. Site study team will provide additional, missing or follow up information in a timely fashion.

# 12 STATISTICS

## 12.1 Statistical Analysis Plan (SAP)

### 12.1.1 Length of therapy

The length of therapy (LOT) is defined as the number of days a patient received an antimicrobial therapy, regardless of dose after enrolment in the study till the completion of the study period (28 days after hospital discharge. It is anticipated that characteristics of the patients recruited in control and experimental groups are equal and proportion test will be performed to confirm that assumption. Patient’s demographic profile and at baseline clinical characteristics will be compared between the control and experimental group.

## 12.2 Description of Statistical Methods

Primary outcome of this study will be analysed by t-test and ANOVA. The mean duration of DOT between control and experimental groups will be considered as primary outcome. Continuous variable will be presented by mean and standard deviation. Categorical variables will be presented by percentage. The primary analysis of this study will be performed by comparing difference of mean duration of DOT in control and experimental group. Mean duration of DOT between control and experimental groups will be tested by t-test and ANOVA. Hospital stay of experimental and control group will be presented. Mortality rate between experimental and control group will be presented by percentage. Mean duration of ICU and general ward stay will be computed and compared between control and experimental groups by t-test and ANOVA. Statistical analyses will be done in Stata version 15 or higher and in R software.

## 12.3 Sample Size Determination

The sample size calculation is based on the hypothesized difference in mean length of antibiotic treatment in days between the experimental and control arm. Based on retrospectively collected data mean length of therapy (LOT) among sepsis patients was 5.2 days with a standard deviation (SD) of 8days. The SD of the duration of antibiotic prescription in population level was derived by dividing the difference between the maximum and minimum value of LOT that was obtained from the retrospective data by four as one of the rules of thumb. The SD is assumed to be the same in the experimental and the control arms. We hypothesize to have a higher mean number of days of LOT in the control group as compared to the experimental group – i.e 5.2 days in the control group vs. 3.2 days in the experimental group, for an absolute reduction of LOT of 2 days. The level of significance was set at 5% (i.e., alpha=0.05). Based on these assumptions and estimating a 80% power, a total of 506participants are required (i.e., 253 in the experimental arm and 253 in the control arm) to verify the study hypothesis. We anticipate a 5% loss to follow and to compensate for this, a total of 532 participants will be enrolled (266 in the experimental arm and 266 in the control arm). The sample size calculations have been performed in R software (version 4.2.1).

## 12.4 Analysis Population

Patients included unblinded by randomization to either the control or experimental arm (procalcitonin-guided antibiotic de-escalation) will be analysed by intention to treat analysis. We will perform an analysis on all participants. Adverse event data between control and experimental arms will be presented by percentage. After completion of D28 follow up of of the first 40 enrolled participants an interim analysis will be done to assess the overall safety of participants.

## 12.5 Stopping Rules

There are no stopping rules adopted in this study. The trial data will be audited at 6 monthly intervals to monitor the participant enrolment, consent, eligibility, and allocation to study groups; adherence to trial interventions and policies to protect participants, including reporting of adverse events; and completeness, accuracy, and timeliness of data collection.

## 12.6 The Level of Statistical Significance

The significance level will be defined at *P* value of <0.05.

## 12.7 Procedure for Accounting for Missing, Unused, and Spurious Data.

The validation rules will include range checks on numeric variables, skip-logic for conditional questions and consistency checks between related variables. Stringent checks will be set on datapoints that are critical to the study outcomes. Cases with missing information on primary outcome variable will not be considered for the analysis and only complete case analysis will be performed. However, missing information on other variables except primary outcome variable will be covered by multiple imputation methods.

## 12.8 Procedures for Reporting any Deviation(s) from the Original Statistical Plan

Proper explanation and justification will be provided in case any deviation required from the original statistical plan to prepare the final report and manuscript.

A general summary of the statistical approaches that will be used is provided in this protocol. A detailed Statistical Analysis Plan (SAP) will be developed as a separate document upon approval of the protocol. All analysis will be performed as per this original Statistical Analysis Plan. The SAP will outline the all the statistical approaches that will be performed together with dummy tables and dummy figures e.g., consort diagram. The main strategy for analysis will follow the intention-to-treat (ITT) principle, which will include all patients according to the group to which they were randomised. In this approach, missing data will be imputed using multiple imputation procedures, as described above. Per-protocol analysis will be done to compliment the ITT analysis. In the per-protocol analysis, all protocol deviation, violations, and patient found to be ineligible post-enrolment will be excluded from this analysis.

As much as practicable, we will maintain the original statistical analysis plan. However, if novel statistical approaches come up in literature, we will be able to adopt the new analysis approaches and reasons for amending the statistical analysis plan will be detailed in the analysis plan and signed by the statistician and the study investigators.

## 12.9 Health Economic Analysis

The cost of antibiotics, procalcitonin assays, cost of hospital stay will be assessed in both arms and compared accordingly.

# 13 DATA MANAGEMENT

The data management aspects of the study are summarised here with details fully described in the Data Management Plan. The data management plan will include:

- Description of the data
- Data collection/generation including study data flow, methodologies for data collection
- Data management, documentation, and curation
- Data security and confidentiality or potential disclosive information
- Analysis and reporting
- Roles and responsibilities
- Study data flow diagram, data collection per time point and study timelines

## 13.1 Source Data

Source documents are where data are first recorded, and from which participants’ CRF data are obtained. These include, but are not limited to, hospital records (from which medical history and previous and concurrent medication may be summarised into the CRF), clinical and office charts, laboratory and pharmacy records, diaries, microfiches, radiographs, and correspondence.

CRF entries will be considered source data if the CRF is the site of the original recording (e.g. there is no other written or electronic record of data). All documents will be stored safely in confidential conditions. On all trial-specific documents, other than the signed consent, the participant will be referred to by the trial participant number/code, not by name.

## 13.2 Access to Data

Direct access will be granted to authorised representatives from the Sponsor, host institution and the regulatory authorities to permit trial-related monitoring, audits, and inspections.

## 13.3 Data Recording and Record Keeping

All trial data will be recorded in the study database via eCRF. The study database will be built in MACRO EDC, a GCP-compliant data management system. The database is password protected and includes internal quality checks to identify data that appear inconsistent, incomplete, or inaccurate. The study data management plan details procedures for data collection and storage, and all activities that will be carried out to ensure security and quality of the data.

Study participants will be identified by a unique trial specific code in the database and on all study documents apart from the signed consent form. The name and any other identifying detail will NOT be included in any trial data electronic file.

Subject records at study site will be stored in binders. At the end of the study, CRFs and essential documents that do not contain patient-identifying information will be scanned and stored electronically. The records will be retained until the youngest child participating in the trial reaches 21 years - or five years following completion of the study, whichever is longer. The study database will be retained indefinitely.

Participant data and results from blood analyses stored in the database may be shared according to the terms defined in the MORU data sharing policy with other researchers to use in the future. Datasets will be de-identified to ensure patient privacy and confidentiality.

# 14 QUALITY ASSURANCE PROCEDURES

## 14.1 Risk assessment

The trial will be conducted in accordance with the current approved protocol, GCP, relevant regulations and standard operating procedures. A risk assessment and monitoring plan will be prepared before the study opens and will be reviewed as necessary over the course of the trial to reflect significant changes to the protocol or outcomes of monitoring activities.

Risks of phlebotomy, peripheral venous catheters, and bladder catheterization:

Blood samples will be taken through an indwelling age-appropriate gauge venous catheter. The catheter will be inserted in the forearm by an experienced physician or nurse using sterile technique. A subcutaneous haematoma can be a complication of this procedure, but this is rare in experienced hands. To minimize the risk of superficial infection and phlebitis, the catheter will be removed after 3-4 days. The relatively small extra amount of blood taken for the study does not cause harm to the patient.

Urine samples will be taken from a urinary catheter or external urine collection bags in patients with coma, incontinence, or in patients in which an indwelling catheter is recommended by the treating team. The main complications of urinary catheterization are tissue trauma and infection. After 48 hours of catheterization, most catheters are colonized with bacteria, thus leading to possible bacteriuria and its complications. To prevent infectious complications, the research physicians will give a not binding advice to the research physicians to remove catheter promptly.

The participants in the control arm could be potentially over-treated with, with associated risks of excess antibiotics, i.e. C. difficile, side effects of medications and development of new AMR etc.

## 14.2 Monitoring

Regular monitoring will be performed by MORU CTSG according to ICH GCP and a monitoring plan. Regular monitoring will be performed according to the trial specific Monitoring Plan. Data will be evaluated for compliance with the protocol and accuracy in relation to source documents as these are defined in the trial specific Monitoring Plan. Following written standard operating procedures, the monitors will verify that the clinical trial is conducted, and data are generated, documented, and reported in compliance with the protocol, GCP and the applicable regulatory requirements. The microbiology culture procedures will be monitored by senior microbiologist based in Chattogram, Bangladesh/ MORU.

## 14.3 Trial committees

### 14.3.1 Trial management committee

Members

1. Prof Arjen M Dondorp, Principal Investigator and Supervisor of DPhil
2. Dr Forhad Uddin Hasan Chowdhury- Co-Principal Investigator and DPhil Student
3. Prof. Aniruddha Ghose Professor of Medicine, Chattogram Medical College Hospital, Bangladesh
4. Prof. Dr. Constance Schultsz, Clinical Microbiologist, Department of Global Health, and Department of Medical Microbiology, Amsterdam University Medical Centres

Netherlands

5. Dr Katherine Plewes, Investigator and co-supervisor of DPhil
6. Dr Luigi Pisani, Investigator and co-supervisor of DPhil
7. Prof. Md. Akram Hossain, Senior Consultant, Microbiology, Molecular Biology and IPC, Apollo Imperial Hospitals
8. Dr Mavuto Mukaka, Chief Statistician and co-supervisor of DPhil
9. Atanu Bhattacharjee Statistician- Statistician
10. Chiraporn Taya - Statistician

Remark: The member may be modified in the future.

Role of trial management committee

1. Monitor all aspects of the trial conduction
2. Monitor all aspects of the trial progress
3. Ensure protocol adherence
4. Ensure quality of trial
5. Take action to safeguard trial participants

Frequency of meeting: Initially 1 monthly for first quarter then quarterly thereafter.

### 14.3.2 Trial steering committee

Members

1. Prof M A Faiz, Professor of Medicine and Former Director General of Health Services, Bangladesh
2. Professor Md. Ahmedul Kabir, Professor of Medicine, and Additional Director General of Health Service Bangladesh
3. Professor Md. Nazmul Islam, Director, Communicable Disease Control Programme (CDC), Government of Bangladesh
4. Satyajit Dhar, Professor, Department of Anaesthesiology and ICU, Chittagong Medical College
5. Professor Dr Ripon Barua, Professor and Head, Department of Microbiology, Chittagong Medical College, Chattogram
6. Dr Ariful Basher, Consultant Infectious Diseases, Infectious Diseases Hospital, Dhaka

Remark: The member may be modified in the future.

The overall task of the Trial Steering Committee is to provide high-level guidance to the trial Management committee for the proper execution of the project. This includes:

1. To monitor the progress of the trial
2. To monitor adherence to the protocol
3. To consider recommendations from the Data Safety Monitoring Board (DSMB)
4. To make recommendations to the Trial Management Committee
5. To make recommendations to the trial Sponsor
6. To consider new information as it becomes available.

The Trial Steering Committee will meet every 6 months, or at an ad hoc basis whenever needed.

### 14.3.3 Independent Data (Safely) Monitoring Committee

An independent Data Safety and Monitoring Board (DSMB) will be set up consisting of qualified volunteers with the necessary knowledge of clinical trials. The DSMB will receive summary reports, prior to each meeting. All data reviewed by the DSMB will be in the strictest confidence. A DSMB charter will outline its responsibilities and how it will operate.

The Data Safety Monitoring Board will consist of:

1. Prof Md Robed Amin, Professor of Medicine and Line Director, Non-Communicable Disease Control Programme, Bangladesh
2. Prof Zaed Hosaain, Professor of Medicine, Dhaka Medical College Hospital
3. Dr. Marc Henrion, Head, Statistical Support Unit, Malawi – Liverpool – Wellcome Programme
4. Dr. Gobinda Banik, Associate Professor of Medicine, Dhaka Medical College Hospital

Remark: The member may be modified in the future

5. Proposed members by the Bangladesh Medical Research Council (BMRC)

Role of the committee

1. To pick up any trends, such as increases in un/expected events, and take appropriate action
2. To seek additional advice or information from investigators if required
3. To evaluate the risk of the trial continuing and take appropriate action where necessary.

The DSMB will conduct a review of all safely after the first 40 patients enrolled, then after 250 patients enrolled and discharged from the hospital and then after total patients enrolled for the duration of the project. The main purpose of the DSMB review will be for the safety of the study and will be performed on all key relevant safety parameters. If a predominance of death, or recurrent infection is noted in the intervention group, or antibiotic related adverse events in the control arm, the study may be stopped based on the DSMB judgement and recommendation with reasons given. This will be included in the DSMB charter.

Unplanned meetings can be held on the initiative of the Medical Monitor (contact details will be summarized in the DSMB Charter and investigators site file), Principal Investigator or DSMB to consider, e.g. SAEs as they are reported OR if an earlier safety review is thought to be indicated.

The principal investigator will submit written DSMB summary reports with recommendations to the ethics committees(s).

# 15 PROTOCOL DEVIATIONS AND VIOLATIONS

A protocol deviation is a variance in a research activity between that described in the protocol that has been reviewed and approved by the IRB/EC and the actual activities or procedures that were performed. A protocol deviation may be classified as minor or significant. A “significant” protocol deviation is also referred to as a protocol violation.

Only the site Investigator may institute emergency departures from the protocol that eliminate an apparent immediate hazard to a subject and that is deemed crucial for the safety and wellbeing of that subject. The site IRB/EC must be made aware of these departures from the protocol in accordance with local requirements.

**Protocol Deviation**

A protocol deviation is any departure from the approved protocol but does not significantly affect the subject’s safety and/or efficacy assessment of the trials (no immediate hazard or data falsification).

Examples of protocol deviations for this protocol include the following:

- Enrolment of a patient after 48 hours of receiving intravenous antibiotic
- Sample collection of procalcitonin 2 hours or later of enrolment
- Failure to perform > 1 consecutive scheduled procalcitonin measurement
- Participant who developed withdrawal criteria during the study but was not withdrawn

**Protocol Violation**

A protocol violation is any departure from the approved protocol that has the potential to or is affecting the safety, rights, and wellbeing of subjects and/or efficacy assessment of the trial.

The following are some examples of protocol violations that should be reported:

- Consent was not obtained prior to entering the patient in the study
- Enrolling a participant who does not meet the study criteria for enrolment (excluding those cases where the investigators have taken the decision to enrol based on the information available at the time of enrolment; should information become available later, e.g., not intended for a parenteral antibiotic)
- Any activity that has the potential to or is affecting the participants’ rights, safety, or welfare
- Error in study randomization, administration, allocation, concealment

# 16 ETHICAL AND REGULATORY CONSIDERATIONS

## 16.1 Declaration of Helsinki

The Investigator will ensure that this trial is conducted in accordance with the principles of the Declaration of Helsinki.

## 16.2 Guidelines for Good Clinical Practice

The Investigator will ensure that this trial is conducted in accordance with relevant regulations and with Good Clinical Practice.

## 16.3 Approvals

The protocol, informed consent form, participant information sheet and any proposed advertising material will be submitted to Oxford Tropical Research Ethics Committee (OxTREC), Bangladesh Medical and Research Council (BMRC), Local Ethics Committee of Chattogram Medical College Hospital for approval.

The Investigator will submit and, where necessary, obtain approval from the above parties for all substantial amendments to the original approved documents.

## 16.4 Other Ethical Considerations

Not applicable

## 16.5 Reporting

The PI shall submit once a year throughout the clinical trial, or on request, an Annual Progress Report to Ethics committees. In addition, an End of Trial notification and final report will be submitted to Ethics committees. After final analysis, results will be presented at a CMCH research meeting to local participation research and clinical members.

## 16.6 Transparency in Research

Prior to the recruitment of the first participant, the trial will have been registered on a publicly accessible trial registry. Results will be uploaded to the registry within 12 months of the end of trial declaration by the PI or their delegate. Where the trial has been registered on multiple public platforms, the trial information will be kept up to date during the trial, and the PI or their delegate will upload results to all those public registries within 12 months of the end of the trial declaration.

## 16.7 Participant Confidentiality

The study will comply with the General Data Protection Regulation (GDPR) and Data Protection Act 2018, which require data to be de-identified as soon as it is practical to do so. The processing of the personal data of participants will be minimised by making use of a unique participant study number only on all study documents and any electronic database(s).  All documents will be stored securely and only accessible by study staff and authorised personnel. The study staff will safeguard the privacy of participants’ personal data.

## 16.8 Expenses and Benefits

Travel for all visits will be reimbursed at a fixed amount of approximately GBP 15.00 to account for transportation cost and lost wages. Prolonged overuse of antibiotics is common clinical practice in Bangladesh. Ultimately this is causing longer hospital stays, antibiotic related side effects, economic burden to patients and hospitals and evolution MDR resistant organisms. Our study will help reduce the overuse of antibiotics which eventually benefit our patients in various ways such as less exposure to antibiotics, less side effects, less cost for treatment to patient shorter duration of hospital stay etc.

# 17 FINANCE AND INSURANCE

## 17.1 Funding

Wellcome Trust of Great Britain

## 17.2 Insurance

The University of Oxford has a specialist insurance policy in place which would operate in the event of any participant suffering harm because of their involvement in the research (Newline Underwriting Management Ltd, at Lloyd’s of London).

## 17.3 Contractual arrangements

Appropriate contractual arrangements will be put in place with all third parties.

# 18 PUBLICATION POLICY

Any data published in the peer-reviewed medical literature will protect the identity of the participants. The trial will be registered in a web-based protocol registration scheme. All those who have made a substantial contribution will be co-authors on publications. Results will be published in peer-reviewed journal articles and relayed to communities in Bangladesh through public meetings and meeting with scientific communities in Bangladesh.

Subjects will be given access to their medical data (such as investigation results) on request. Study results will be available on request from the Principal Investigator, once data analysis and report writing are completed.

# 19 ARCHIVING

At the end of the study, CRFs and essential documents that do not contain patient-identifying information will be scanned and stored electronically. The records will be retained until the youngest child participating in the trial reaches 21 years - or five years following completion of the study, whichever is longer. The study database will be retained indefinitely.

# 20 APPENDIX A: TRIAL FLOW CHART

# 21 APPENDIX B: SCHEDULE OF SAMPLING AND PROCEDURES

| **Procedures** | **Visits** | | | | |
| --- | --- | --- | --- | --- | --- |
|  | **Day 0** | | **Daily till Discharge** | **After 28 days of discharge** | **Suspected recurrent infection** |
|  | **Screening** | **Baseline** |  |  |  |
| Informed consent | X |  |  |  |  |
| Demographics |  | X |  |  |  |
| Medical history |  | X |  |  |  |
| Concomitant medications (including antibiotics) |  | X | X | X |  |
| Physical examination |  | X |  |  |  |
| Vital signs  (clinical assessments) | X | X | X | X (if presents physically) | X |
| Clinical history questionnaire |  | X |  |  | X |
| Complications questionnaire |  | x | x |  |  |
| Symptom questionnaire on follow up |  |  |  | X |  |
| Laboratory tests (basic biochemical tests such as CBC, , S. creatinine, S. Bilirubin etc |  | X |  |  |  |
| Urinalysis |  | x |  |  | x |
| PCT assay (intervention group) |  | X | X |  |  |
| Blood or other specimen culture |  | X |  |  | x |
| Eligibility assessment | X |  |  |  |  |
| Randomisation | X |  |  |  |  |
| Dispensing of trial drugs | NA |  |  |  |  |
| Compliance |  |  | X |  |  |
| Adverse event assessments |  |  | X |  |  |

**Remark**

**Baseline**

- Imaging (a systematic 12-zone lung ultrasound) will be done if clinically indicated on all study participants at baseline (D0H0) following international guidelines.

**Daily**

- Daily symptom questionnaire for complications or clinical deterioration/recurrent infection.

- GCS, blood pressure, heart rate, capillary refill time, respiratory rate, and oxygen saturation, perfusion index, temperature (aural) daily up to discharge. Vital signs will be repeated if clinical deterioration/recurrent infection is suspected.

# 22. APPENDIX C: AMENDMENT HISTORY

| **Amendment No.** | **Protocol Version No.** | **Date issued** | **Author(s) of changes** | **Details of Changes made** |
| --- | --- | --- | --- | --- |
| 1 | 2.0 | 23 May 2023 | Forhad Uddin Hasan Chowdhury, Co- principal investigator | - Addition of Professor Philippe Guerin as co-investigator of the study and Dr Marc Henrion as a member of the DSMB  - Analysis of the preliminary data informing the sample size was refined to make sure it is aligned with the “length of therapy” primary endpoint. This resulted in an increase of the total sample size from 466 to 532 participants  - Plasma procalcitonin (PCT) test is replaced with serum PCT test because it gives more accurate results.  - As required by the National Research Ethics Committee, the working and operational definition for sepsis have been added  -One exclusion criterion was rephrased while two were deleted due to redundancy |
| 2 | 3.0 | 31 January 2024 | Forhad Uddin Hasan Chowdhury, Co- principal investigator | -Change of Clinical phase of the study to Phase 3 from proof of concept as suggested by the DSMB Members  - add clinical phase 3 to the study title  ***Objectives***  - **Change in the time point of primary objective**  **Length of antibiotic treatment:** number of days of antibiotic treatment during the study period (from during hospitalisation)  **Changes in the secondary objectives**  -Comparison of Defined Daily Dose (DDD) data with the pre-study data will not be assessed. Only DDD between two arms will be compared.  - **Length of antibiotic treatment (LOT) during hospitalisation period:** number of days of antibiotic treatment during the hospitalisation period (primary objective in previous)  ***Intervention***  (corrected in the text and an illustration of the trial intervention is attached as flow chart)  PCT assays: In patients will high PCT the target PCT will be counted as 80% reduction from the peak value (not from baseline value)  Baseline PCT value 0.5ng/ml- testing of PCT to consecutive three from only baseline assay and providing non-binding advice for this group at 48 hours then 72 hours  Consort diagram is attached as image.  ***Clinical assessment during hospitalisation***  a capillary blood glucose will be measured at baseline and if clinically indicated during follow up  ***Sample handling***  A corrected amount of blood required for tests and changes in the storage of specimens.  ***SAEs***  SAEs will be reported as per CTACE grading.  ICU referral is added as an adverse event and any deaths irrespective of relation to the study intervention or disease complication or treatment complication will be reported as SAE  ***Adding of supervisor***  Professor Mavuto Mukaka has been added as a co-supervisor.  ***Committees***  Trial steering committee and DSMB have been updated.  ***Protocol deviation***  Failure to perform > 1 consecutive scheduled procalcitonin measurement will be considered as protocol deviation  ***Removal of appendix C***  The tables of antibiotic therapies have been removed since the treating team uses an app to fix the antibiotic duration. |

# 23.REFERENCES

, ATC/DDD Index 2023, WHO" Dec 19, ", 2022-last update [20/12/, 2022].

*Standard Treatment Guidelines (STG) on Antibiotic Use in Common Infectious Diseases of Bangladesh.* 2021. CDC, DGHS, Bangladesh.

AHMED, I., RABBI, M.B. and SULTANA, S., 2019. Antibiotic resistance in Bangladesh: A systematic review. *International Journal of Infectious Diseases,* **80**, pp. 54-61.

BOUADMA, L., MD, LUYT, C., MD, TUBACH, F., MD, CRACCO, C., MD, ALVAREZ, A., MD, SCHWEBEL, C., MD, SCHORTGEN, F., MD, LASOCKI, S., MD, VEBER, B., Prof, DEHOUX, M., MD, BERNARD, M., MD, PASQUET, B., MS, RÉGNIER, B., Prof, BRUN-BUISSON, C., Prof, CHASTRE, J., Prof and WOLFF, M., Prof, 2010. Use of procalcitonin to reduce patients' exposure to antibiotics in intensive care units (PRORATA trial): a multicentre randomised controlled trial. *The Lancet (British edition),* **375**(9713), pp. 463-474.

DALTON, B.R., MACTAVISH, S.J., BRESEE, L.C., RAJAPAKSE, N., VANDERKOOI, O., VAYALUMKAL, J. and CONLY, J., 2015. Antimicrobial use over a four-year period using days of therapy measurement at a Canadian pediatric acute care hospital. *Canadian Journal of Infectious Diseases and Medical Microbiology,* **26**(5), pp. 253-258.

DANDONA, P., NIX, D., WILSON, M.F., ALJADA, A., LOVE, J., ASSICOT, M. and BOHUON, C., 1994. Procalcitonin increase after endotoxin injection in normal subjects. *The Journal of Clinical Endocrinology & Metabolism,* **79**(6), pp. 1605-1608.

DAVIES, J., 2015. Procalcitonin. *Journal of clinical pathology,* **68**(9), pp. 675-679.

DE JONG, E., VAN OERS, J.A., BEISHUIZEN, A., VOS, P., VERMEIJDEN, W.J., HAAS, L.E., LOEF, B.G., DORMANS, T.C., VAN MELSEN, G.C., KLUITERS, Y.C., KEMPERMAN, H.J., VAN DEN ELSEN, M.J., SCHOUTEN, J.A., STREEFKERK, J.O., KRABBE, H.G., KIEFT, H., KLUGE, G.H., VAN DAM, V.C., VAN PELT, J., BORMANS, L., OTTEN, M.B., REIDINGA, A.C., ENDEMAN, H., TWISK, J.W., VAN DE GARDE, EWOUDT M W, DE SMET, ANNE MARIE G A, KESECIOGLU, J., GIRBES, A.R., NIJSTEN, M.W. and DE LANGE, D.W., 2016. *Efficacy and safety of procalcitonin guidance in reducing the duration of antibiotic treatment in critically ill patients: a randomised, controlled, open-label trial.* Elsevier BV.

DELIBERATO, R.O., MARRA, A.R., SANCHES, P.R., DALLA VALLE MARTINO, M., DOS SANTOS FERREIRA, C.E., PASTERNAK, J., PAES, A.T., PINTO, L.M., DOS SANTOS, O.F.P. and EDMOND, M.B., 2013. Clinical and economic impact of procalcitonin to shorten antimicrobial therapy in septic patients with proven bacterial infection in an intensive care setting. *Diagnostic microbiology and infectious disease,* **76**(3), pp. 266-271.

DONDORP, A.M., LIMMATHUROTSAKUL, D. and ASHLEY, E.A., 2018. What’s wrong in the control of antimicrobial resistance in critically ill patients from low- and middle-income countries? *Intensive Care Medicine,* **44**(1), pp. 79-82.

JENSEN, J.U., HEIN, L., LUNDGREN, B., BESTLE, M.H., MOHR, T.T., ANDERSEN, M.H., THORNBERG, K.J., LØKEN, J., STEENSEN, M., FOX, Z., TOUSI, H., SØE-JENSEN, P., LAURITSEN, A.Ø, STRANGE, D., PETERSEN, P.L., REITER, N., HESTAD, S., THORMAR, K., FJELDBORG, P., LARSEN, K.M., DRENCK, N.E., ØSTERGAARD, C., KJÆR, J., GRARUP, J. and LUNDGREN, J.D., 2011. Procalcitonin-guided interventions against infections to increase early appropriate antibiotics and improve survival in the intensive care unit: A randomized trial. *Critical care medicine,* **39**(9), pp. 2048-2058.

LEE, C., KWA, A.L.H., APISARNTHANARAK, A., FENG, J., GLUCK, E.H., ITO, A., KARUNIAWATI, A., PERIYASAMY, P., PRATUMVINIT, B., SHARMA, J., SOLANTE, R., SWAMINATHAN, S., TYAGI, N., VU, D.M., ZIRPE, K. and SCHUETZ, P., 2020. *Procalcitonin (PCT)-guided antibiotic stewardship in Asia-Pacific countries: adaptation based on an expert consensus meeting.* Walter de Gruyter GmbH.

LLOR, C. and BJERRUM, L., 2014. Antimicrobial resistance: risk associated with antibiotic overuse and initiatives to reduce the problem. Ther Adv drug Saf.

MARUNA, P., NEDELNÍKOVÁ, K. and GÜRLICH, R., 2000. Physiology and genetics of procalcitonin. *Physiological research,* **49 Suppl 1**, pp. S57-S61.

NEWS, 2020. *National Early Warning Score systems that alert to deteriorating adult patients in hospital Medtech innovation briefing.* National Institute of Science and Healthcare Excellence.

NOBRE, V., HARBARTH, S., GRAF, J., ROHNER, P. and PUGIN, J., 2008. Use of Procalcitonin to Shorten Antibiotic Treatment Duration in Septic Patients: A Randomized Trial. *American journal of respiratory and critical care medicine,* **177**(5), pp. 498-505.

OJUAWO, O.B., FAWIBE, A.E., DESALU, O.O., ALADESANMI, A.O., OJUAWO, A.B., OPEYEMI, C.M., JIMOH, A.O. and SALAMI, A.K., 2021. Clinical utility of serum procalcitonin in adult patients admitted with community-acquired pneumonia in Ilorin, Nigeria. *Journal of the Pan African Thoracic Society,* **2**, pp. 77-84.

O'NEILL, J., 2016. *Tackling drug-resistant infections globally: final report and recommendations.* Government of the United Kingdom.

PAIRATTANAKORN, P., ANGKASEKWINAI, N., SIRIJATUPHAT, R., WANGCHINDA, W., TANCHAROEN, L. and THAMLIKITKUL, V., 2021. Diagnostic and Prognostic Utility Compared Among Different Sepsis Scoring Systems in Adult Patients With Sepsis in Thailand: A Prospective Cohort Study. *Open forum infectious diseases,* **8**(1), pp. ofaa573.

POKHAREL, S., RAUT, S. and ADHIKARI, B., 2019. Tackling antimicrobial resistance in low-income and middle-income countries. *BMJ Global Health,* **4**(6), pp. e002104.

SAMSUDIN, I. and VASIKARAN, S.D., 2017. Clinical utility and measurement of procalcitonin. *The Clinical Biochemist Reviews,* **38**(2), pp. 59.

SCHUETZ, P., CHRIST-CRAIN, M., THOMANN, R., FALCONNIER, C., WOLBERS, M., WIDMER, I., NEIDET, S., BLUM, C., FRICKER, T., SCHILD, U., REGEZ, K., SCHOENENBERGER, R., HENZEN, C., BREGENZER, T., KRAUSSE, M., HOESS, C., BUCHER, H., ZIMMERLI, W. and MUELLER, B., 2009. Effect of procalcitonin-based guidelines compared with standard guidelines on antibiotic use in lower respiratory tract infections: the randomized-controlled multicenter ProHOSP trial. *Critical Care,* **13**(Suppl 1), pp. P386.

SCHULZ, K.F., ALTMAN, D.G. and MOHER, D., 2010. CONSORT 2010 statement: updated guidelines for reporting parallel group randomised trials. *Journal of Pharmacology and pharmacotherapeutics,* **1**(2), pp. 100-107.

SIMON, L., GAUVIN, F., AMRE, D.K., SAINT-LOUIS, P. and LACROIX, J., 2004. Serum Procalcitonin and C-Reactive Protein Levels as Markers of Bacterial Infection: A Systematic Review and Meta-analysis. *Clinical infectious diseases,* **39**(2), pp. 206-217.

STOP AMR, 2020. *AMR impacts 12 of 17 UN SDGs, but not mentionned in any way. Why?* .

TAN, T.L., KANG, C.W., OOI, K.S., TAN, S.T., AHMAD, N.S., NASURUDDIN, D.N., ITHNIN, A., TAJUL ARIFIN, K., HENG, L.Y. and HASSAN, N.I., 2021. Comparison of sPLA2IIA performance with high-sensitive CRP neutrophil percentage PCT and lactate to identify bacterial infection. *Scientific Reports,* **11**(1), pp. 1-9.

WACKER, C., PRKNO, A., BRUNKHORST, F.M. and SCHLATTMANN, P., 2013. Global extent of chloroquine-resistant Plasmodium vivax: a systematic review and meta-analysis. *The Lancet Infectious Diseases,* **13**(5), pp. 426.

WHO, 2020-last update, The fight against Antimicrobial Resistance is closely linked to the Sustainable Development Goals [03/12/, 2022].

WORLD BANK, 2017. *Drug-Resistant Infections : A Threat to Our Economic Future.* World Bank, Washington, DC.

stylefix
